# Gene expression in primate liver during viral hemorrhagic fever

**DOI:** 10.1186/1743-422X-6-20

**Published:** 2009-02-12

**Authors:** Mahmoud Djavani, Oswald R Crasta, Yan Zhang, Juan Carlos Zapata, Bruno Sobral, Melissa G Lechner, Joseph Bryant, Harry Davis, Maria S Salvato

**Affiliations:** 1 Institute of Human Virology, University of Maryland School of Medicine, Baltimore, MD 21201, USA; 2 Virginia Bioinformatics Institute at Virginia Tech, Blacksburg, VA 24061, USA; 3 University of Southern California, Keck School of Medicine, Los Angeles, CA 90089, USA

## Abstract

**Background:**

Rhesus macaques infected with lymphocytic choriomeningitis virus (LCMV) provide a model for human Lassa fever. Disease begins with flu-like symptoms and progresses rapidly with fatal consequences. Previously, we profiled the blood transcriptome of LCMV-infected monkeys (M. Djavani et al J. Virol. 2007) showing distinct pre-viremic and viremic stages that discriminated virulent from benign infections. In the present study, changes in liver gene expression from macaques infected with virulent LCMV-WE were compared to gene expression in uninfected monkeys as well as to monkeys that were infected but not diseased.

**Results:**

Based on a functional pathway analysis of differentially expressed genes, virulent LCMV-WE had a broader effect on liver cell function than did infection with non-virulent LCMV-Armstrong. During the first few days after infection, LCMV altered expression of genes associated with energy production, including fatty acid and glucose metabolism. The transcriptome profile resembled that of an organism in starvation: mRNA for acetyl-CoA carboxylase, a key enzyme of fatty acid synthesis was reduced while genes for enzymes in gluconeogenesis were up-regulated. Expression was also altered for genes associated with complement and coagulation cascades, and with signaling pathways involving STAT1 and TGF-β.

**Conclusion:**

Most of the 4500 differentially expressed transcripts represented a general response to both virulent and mild infections. However, approximately 250 of these transcripts had significantly different expression in virulent infections as compared to mild infections, with approximately 30 of these being differentially regulated during the pre-viremic stage of infection. The genes that are expressed early and differently in mild and virulent disease are potential biomarkers for prognosis and triage of acute viral disease.

## Background

Arenaviruses are rodent-borne viruses that can be transmitted to primates, occasionally causing lethal hemorrhagic fever. Arenaviruses causing Lassa fever and South American hemorrhagic fevers have been classified as Category A bio-threats in the United States because of their virulence. Human beings infected with a hemorrhagic fever virus initially exhibit flu-like symptoms, and disease progresses so rapidly that diagnosis and appropriate treatments are often too late. Laboratory studies using the arenavirus lymphocytic choriomeningitis virus (strain LCMV-WE) showed that rhesus macaques develop an acute viral disease similar to Lassa fever in human beings [1-8]. LCMV-associated hemorrhagic fever in macaques provided a practical model for disease in a well-controlled laboratory environment. Whereas LCMV-WE was highly pathogenic for primates and guinea pigs, animals infected with the Armstrong strain (LCMV-ARM) did not manifest disease or viremia and were protected from lethal challenge with LCMV-WE [8].

Our previous publications on the pathology of LCMV-WE infection described up-regulation of liver gene expression related to organ development, regeneration and inflammatory responses [2,3,5]. Blood profiles of LCMV-infected macaques revealed distinct pre-viremic and viremic stages of infection, with over 90 virulence-specific gene-expression changes detectable before the viremic stage [3]. The viremic or symptomatic stage of the virulent infection was characterized by high viral loads, high liver enzymes, thromocytopenia, high plasma levels of IP-10, IFN-γ, MCP-1, IL-6, TNFRI and TNFRII, as well as clinical signs of appetite loss, withdrawal, and fever [2-6,8]. Diseased liver tissue had disorganized parenchyma and mononuclear infiltrates (infiltrates were also seen in lung), whereas tissue from animals that were infected but not diseased had no infiltrates and appeared healthy [4-6]. Gene expression of PBMC was remarkable for its down-regulation of several signaling pathways, e.g. via IL-1β receptor, epithelial growth factor receptor, and retinoic acid receptor [3] and this decrease was corroborated by studies in a guinea pig model for Lassa fever [9,10]. A dramatic and early drop in cyclo-oxygenase-2 gene (PTGS2) expression was observed in the primate model that could directly account for the drop in prostacyclin and platelet dysfunction described in Lassa fever [11-13].

Despite the complex clinical presentation of viral hemorrhagic fever, we chose to focus on liver gene expression because that organ had the highest virus titers. Liver tissue contains several cell types, and approximately 25% of the changes in transcriptome do not result in proteomic changes [14]; so with these *caveats *in mind, we examine the most prominent transcriptome changes in relation to published information about primate liver infections. Down-regulated genes involved in fatty acid synthesis and up-regulated genes involved in gluconeogenesis presented a profile that has been associated with starvation and also typifies LCMV infection of macaques. Although most of the gene expression changes controlling intermediary metabolism could be categorized as general homeostatic responses to infection, some gene-expression changes, such as in transcripts related to amino-acid catabolism and protein phosphatase, were strongly associated with an early virulent profile and were more likely to contribute to fatal disease. Approximately 30 genes were identified as potential signature biomarkers for the onset of virulent LCMV-related liver disease. We discuss those gene expression changes that are similar to other viral diseases of liver and those changes that seem unique to an acute arenavirus infection.

## Materials and methods

### Experimental samples

Twenty healthy adult rhesus macaques, five to nine years of age, were used for a terminal study [3]. Liver tissue samples were obtained on the day of euthanasia, from uninfected controls and infected animals. Before euthanasia, blood was taken for clinical chemistry and hematology; tissues were processed for RNA extraction within 15 minutes after collection.

LCMV infection of rhesus macaques was described previously [3]. Briefly, animals were either uninfected or infected intravenously (i.v.) with LCMV-ARM or LCMV-WE using 10^3 ^plaque forming units (pfu) virus. LCMV-WE alone, at 10^3 ^pfu i.v. is uniformly lethal. Four animals infected with both LCMV-ARM and LCMV-WE (10^3 ^pfu each), did not develop symptoms and were classified as "infected-but-not-diseased". Infection in macaques was monitored by plaque assay of infectious particles in plasma, by infectious center assay of PBMC and liver tissues, and by RT-PCR to detect viral RNA in tissues [3].

### RNA preparation and GeneChip hybridization

Total RNA was prepared from LCMV-infected or uninfected liver tissues following TRIzol (GIBCO-BRL) extraction and purification using an RNeasy system according to the manufacturer's instructions (QIAGEN, Valencia, CA). A QIAGEN RNase-free DNase supplement kit was used to ensure that the RNA had no DNA contamination. All RNA samples were checked for both quality and quantity as described previously [3]. RNA that passed this initial quality control screen was then labeled according to the standard target labeling protocols provided by Affymetrix and hybridized to the GeneChip human genome U133 Plus 2.0 array (Affymetrix, Santa Clara, CA) as described by the manufacturer . The use of the Affymetrix human genome microarrays for monitoring transcriptome changes in rhesus macaque tissues has been validated by other studies [15-17].

### Microarray data analysis

Microarray data analyses were performed using the Array Data Analysis and Management System (ADAMS), currently being developed at VBI . The system uses publicly available tools for analysis of the data. Briefly, raw probe intensities were normalized and summarized using a robust multichip average of G+C content algorithm (gcRMA algorithm) [18]. Detection calls (present, marginal, or absent) for each probe set were obtained using the mas5calls function in the Affy R package [19]. For paired comparisons, only genes with at least one present call among the compared samples were included.

A total of 20 samples were used to generate the microarray data . Data from the 20 samples were grouped as follows to perform statistical analyses: uninfected controls (three samples), samples infected with the virulent strain LCMV-WE taken during the pre-viremic stage (four samples), samples infected with LCMV-WE during the viremic stage (five samples), samples infected with LCMV-WE during the post-viremic stage (two samples), and six samples that were infected but not diseased (two monkeys infected with LCMV-ARM and four infected with both LCMV-ARM and LCMV-WE) (Table 1). Cluster analysis justified grouping LCMV-ARM samples with LCMV-ARM+WE samples since heat maps of gene expression from LCMV-ARM-infected animals were most similar to those from animals doubly-infected with LCMV-ARM and LCMV-WE (see Additional File 1). Mean *n*-fold changes were calculated using a division of normalized expression values between experimental sample and uninfected control. False discovery rates [20] of the pair-wise comparisons were calculated using *p*-values from the Linear Models for Microarray Data (LIMMA) package [designed for analysis of Affymetrix array data; ]. Differentially-regulated genes were selected using a 2-fold cut-off and a false discovery rate (FDR) of < 0.05.

**Table 1:** Macaque liver tissues used for transcriptome analyses

**Liver samples**	**AST/ALT^d^**	**Glucose^e^**	**Triglycerides^f^**	**Virus titer^g^**
**Uninfected controls**				
Rh Ctrl7	38/65	74	65	<10^2^
Rh Ctrl8	24/53	79	61	<10^2^
Rh Ctrl9	47/71	87	77	<10^2^
**LCMV-WE1-Pre-viremic**				
Rh 1WE (day 1)^a^	44/61	**53**	**27**	<10^2^
Rh 2WE (day 1)	63/54	58	**30**	<10^2^
Rh 3WE (day 2)	59/37	60	**18**	<10^2^
Rh 5WE (day 3)	27/71	62	**37**	<10^2^
**LCMV-WE2-Viremic**^b^				
Rh 6WE (day 4)	**96/137**	71	**33**	<10^2^
Rh 7WE (day 4)	24/38	67	**18**	<10^2^
Rh 8WE (day 6)	26/56	**54**	**32**	**1.4 × 10**^4^
Rh 9WE (day 6)	**125/82**	70	**31**	**5.0 × 10**^3^
Rh 10WE (day 7)	**190/325**	66	**30**	**1.5 × 10**^6^
**LCMV-WE3 Terminal**				
Rh 11WE (day 11)	**642/587**	**30**	**235**	**5.5 × 10**^5^
Rh 12WE (day 12)	**1504/681**	**40**	**1402**	**1.0 × 10**^6^
**LCMV-not diseased**^c^				
Rh 3ARM (day 3)	45/74	70	75	<10^2^
Rh 5ARM (day 5)	18/40	78	62	<10^2^
Rh 1ARM/WE-1	52/24	65	79	<10^2^
Rh 1ARM/WE-5	Not done	Not done	ND	<10^2^
Rh 2ARM/WE-6	56/34	63	80	<10^2^
Rh 2ARM/WE-2	Not done	Not done	ND	<10^2^

^a ^(day 1) is the day of necropsy after infection. Each sample is from the liver of a different rhesus macaque.

^b ^Pre-viremic means liver was harvested day 1, day 2 or day 3 after infection; Viremic means liver was harvested day 4 to day 7, and Terminal means liver was harvested day 11 or 12 during the terminal stage of disease. ND = Not done.

^c ^Animals that were LCMV-infected but not diseased were either infected with LCMV-ARM and sacrificed day 3 or day 5 after infection, or they were infected with LCMV-ARM and LCMV-WE (10^3 ^pfu each virus) and sacrificed on day 30 after infection.

^d ^AST/ALT means the ratio of plasma levels of aspartate aminotransferase to alanyl aminotransferase in international units per liter (IU/L). Normal ranges are AST/ALT = 18–82/22–87 IU/L. **Values outside the reference range are bolded**.

^e ^Serum glucose levels are in mg/dL with a reference range of 55–80 mg/dL (Animal Diagnostics Laboratory, Baltimore, MD.)

^f ^Serum triglyceride levels have a reference range of 50–82 mg/dL. Other parameters derived from blood analysis include red blood cells, hemoglobin levels and cholesterol levels which were all within normal ranges for all samples.

^g ^Virus was titered on Vero cell monolayers as described [2], and given as plaque forming units per gram of liver tissue. Samples in the viremic group that had undetectable titers (<10^2 ^pfu/gm) were positive for virus nucleic acid by RT-PCR and virus co-cultivation as described previously [3].

Pairwise comparisons were performed to identify 3,125 differentially-regulated genes (*n*-fold of at least 2 and FDR P < 0.05) between infected samples (*n *= 9) and uninfected control samples (*n *= 3) or between infected samples (*n *= 9) and samples from animals that were infected but not diseased (*n *= 6). Six separate pair-wise comparisons were also performed to compare the three groups of infected samples: pre-viremic (*n *= 4), viremic (*n *= 5) and post-viremic (*n *= 2) to the two groups; controls (*n *= 3) or non-diseased samples (*n *= 6). These six pairwise comparisons resulted in identification of 4,482 differentially-regulated genes (*n*-fold of at least 2 and FDR P < 0.05) amongst any one pairwise comparison. KEGG software was used to identify key functions and metabolic pathways differentially-regulated between uninfected and LCMV-infected liver samples. The raw gene expression data and the related experimental information from this study can be found at , platform number GSE12254.

### Quantitative real-time reverse-transcriptase PCR

Genes for microarray analysis were validated by quantitative real-time PCR as we described [3] to determine the extent to which gene expression was up- or down-regulated as a result of infection. Monkey and human-specific primers were used to validate expression levels for selected host genes. Although we ordinarily used primers derived from GAPDH, Actin, or 18S RNA for baseline expression, we found that the expression of these three genes varied in infected versus uninfected liver, so we searched for genes that were expressed in liver but at a steady level for all infection time-points. We found that expression of the protein kinase C substrate 80 K-H gene (PRKCSH, or hepatocystin) had a less than 0.04-fold standard deviation over all the samples used for this liver transcriptome analysis. Baseline expression of PRKCSH could be determined with primers for the *Macaca mulatta *sequence (first, CGTTAGGCAGCCGTGC and second, GGCCGTGGAGGTCAAGAGGC).

## Results

### Identification of differentially-regulated genes in LCMV-infected rhesus macaque liver

Liver RNA from LCMV-infected macaques was used for transcriptome profiling with the goal of identifying gene expression that differentiated infected from uninfected liver and that differentiated virulently-infected (diseased) from mildly-infected (non-diseased) liver. As described previously [3], we characterized two major stages of infection, pre-viremic (day 1 to day 3) and viremic (day 4 to day 7) and compared them to uninfected samples or samples from monkeys infected with LCMV that did not have disease. Three types of results are presented here: 1) genes with the greatest differential expression after pair-wise comparisons of infected and uninfected liver samples, 2) gene expression associated with major pathways in the liver such as gluconeogenic, glycolytic, lipogenic and coagulation pathways, and 3) genes with the greatest differential expression after pair-wise comparison of virulent and mildly-infected samples. In the latter category we especially noted gene expression changes that occurred before the viremic stage of disease, and could potentially serve as biomarkers that discriminate between virulent and benign infections.

**Figure 1:**
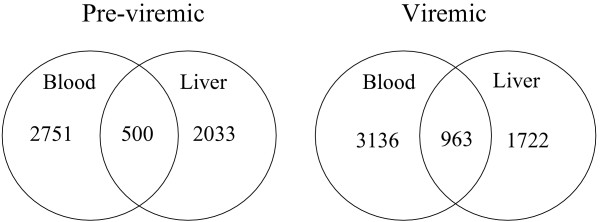
**Venn diagram of gene expression in virulently-infected liver compared to virulently-infected blood**. Genes identified as being differentially-regulated in both blood and liver can be found in Additional Files 2, 3, 4, 5, 6, 7.

The liver is highly vascularized and sensitive to changes in blood composition. Since liver tissue contains a mixture of cells including hematopoietic cells found in the circulation, we examined the overlap of gene expression in liver and in blood for the virulently-infected monkeys (Figure 1; Additional Files 2, 3, 4, 5, 6, 7). Among the subset of genes displaying comparably elevated expression in blood and liver were interferon-induced transmembrane protein 1 (IFITM1), tumor necrosis factor (ligand) superfamily, member 10 (TNFSF10, or TRAIL), ubiquitin-conjugating enzyme E2L6 (UBE2L6), BCL2-related protein A1 (BCL2A1), profilin 1 (PFN1), some coagulation pathway genes, and some JAK-STAT/toll-like receptor signaling pathway genes.

Additional File 8 lists differentially-regulated genes in macaque liver (n = 4482), which include 1234 significantly (*p *< 0.05) up-regulated genes and 123 down-regulated genes (the remainder were up-regulated at some time points and down-regulated at others). Differential gene expression is presented in two ways: 1) LCMV-WE-infected (diseased) liver compared to uninfected liver, and 2) LCMV-WE-infected (diseased) liver compared to LCMV-infected non-diseased liver. Differentially-expressed genes were defined as those that had at least a 2-fold alteration in LCMV-infected livers compared to the uninfected controls.

**Figure 2:**
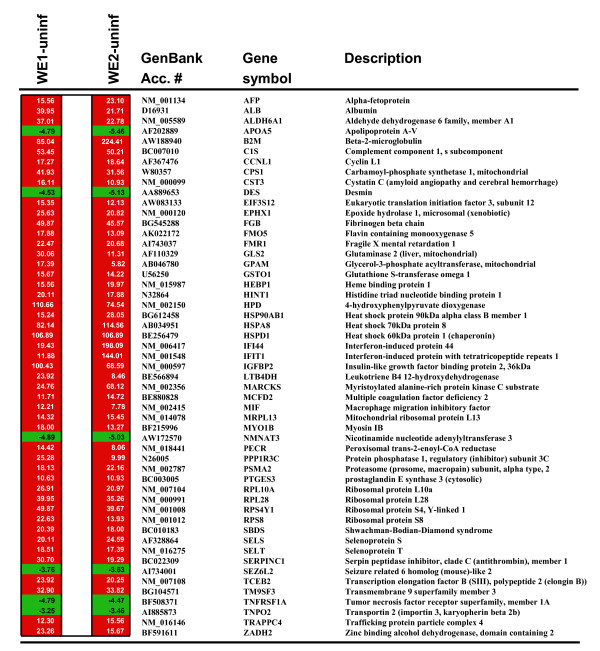
**The most differentially regulated genes in liver after infection of macaques with LCMV-WE**. WE1 refers to pre-viremic samples (day 1–3) and WE2 refers to viremic samples (day 4–7). Both WE1 and WE2 are compared with uninfected samples (WE1 vs uninf) using the sample numbers shown in Table 1. Nfold refers to fold up- or down- regulation of each gene expressed in a virulently-infected liver in relation to expression in an uninfected liver. The *p *values for these genes are all less than 0.05. This is a subset of Additional File 8.

Genes with the highest differential expression between LCMV-infected and uninfected livers (**53 are listed in **Figure 2) encode several heat shock proteins, ribosomal proteins, energy-generating enzymes, and proteins known to be involved in anti-microbial defense [e. g. Shwachman-Bodian-Diamond syndrome (SBDS), complement (C1S), and interferon-inducible genes (IFITM1, IFITM2)]. Prominent genes associated with energy-generation include HPD and GSL2 encoding enzymes that break down amino acids. Also notable are two gene products with anti-inflammatory functions: 1) HSP60 (encoded by HSPD1) that down-regulates T-bet, NF-kappaB, and NFAT and up-regulates GATA-3, leading to decreased secretion of TNF-alpha [21], and 2) LTB4DH that encodes a dehydrogenase which inactivates several eicosanoids such as leukotriene B4 [22].

### Metabolic pathway gene expression in LCMV-infected rhesus macaques

To understand the metabolic responses to LCMV infection, changes in gene expression in macaque livers were examined in relation to their associated metabolic pathways using the KEGG pathways analysis. Additional File 9 shows 32 of the most significantly affected pathways in LCMV-infected macaque liver with respect to uninfected liver for both pre-viremic and viremic stages of infection. Four of these pathways are described here and shown in (Tables 3,4,5 and 6).

**Table 2:** Validation of microarray results by real-time PCR^a^

**Gene Symbol**	WE vs uninfected		WE vs no disease	
	
(GenBank ID)	Pre-viremic	Viremic Stage	Pre-viremic	Viremic stage
**ACACB **(AI057637)	-3.58/-5	-4.38/-5	-2.16/-2	-2.64/-2
**ACVR1 **(NM_001105)	10.85/15	7.62/12	7.46/11	5.24/10
**ARG2 **(U75667)	1.02/2	1.04/1	-12.13/-9	-11.88/-5
**C1S **(BC007010)	53.45/25	50.21/22	6.73/4.2	6.32/3.7
**CYP26A1**(NM_000783)	9.85/13	2.11/1.2	10.56/14	2.27/2.9
**FGB **(BG545288)	49.87/32	45.57/29	3.36/2.4	3.07/2.0
**FST **(NM_013409)	-1.67/1.8	1.09/1.3	-16.8/-19	-9.25/-16
**GOT1 **(BC000498)	3.92/3.0	2.0/1.6	-6.54/-13	-12.82/-18
**HBA1/HBA2 **(V00489)	2.79/2	1.2/3.1	-12.91/-11	-30.06/-25
**HSPB1 **(NM_001540)	28.64/22	36.76/43	9.38/7.2	12.04/9.7
**IFI44 **(NM_006417)	19.43/14	198.1/166	3.71/4.0	37.79/21
**INHBA **(M13436)	1.71/1.9	-1.13/1.3	11.16/15	5.78/8.7
**PPARGC1A **(NM_013261)	-1.22/1.0	3.01/1.9	-21.41/-9	-5.82/-3
**PPP1R3C **(N26005)	25.28/32	9.99/12	156.5/87	61.82/45
**SDS **(NM_006843)	-1.97/1.7	-2.07/1.2	-32.22/-28	-33.82/-29
**TGFBR1 **(AA604375)	4.29/4.8	3.1/2.5	1.33/1.6	-1.04/1.5
**PRKCSH **(AI815793)	<2/-^b^	<2/-	<2/-	<2/-

^**a **^The numbers represent N-folds from Affychip data (most in Figure 4, all in Additional file 8) and N-fold by real-time PCR (N_affy_/N_PCR_). N-folds by real-time PCR make use of liver RNA from uninfected, pre-viremic LCMV-WE-infected, viremic LCMV-WE-infected, and infected-but-not-diseased monkeys.

^**b **^PRKCSH is the gene symbol for protein kinase C substrate 80 K-H to which the PCR data are normalized. It is expressed in liver and has a less than 0.04 standard deviation over all samples used for this liver transcriptome analysis. (-) means it is the reference value.

**Table 3:** Expression of genes involved with TCA cycle and glucose metabolism in LCMV-WE infected macaque liver

			**Pre-viremic**		**Viremic**	
				
**GenBank Accession no.**	**Symbol**	**Gene description**	**Fold change^a^**	***p*^b^**	**Fold change^a^**	***p*^b^**
***Citric acid cycle***						
NM_001096	**ACLY**	ATP citrate lyase	2.14	0.0809	3.58	0.0040
AI363836	**FH**	Fumarate hydratase	5.54	0.0045	3.05	0.0356
AI826060	**IDH3A**	Isocitrate dehydrogenase 3 (NAD+) alpha	1.51	0.4150	2.29	0.0440
AF023266	**IDH3B**	Isocitrate dehydrogenase 3 (NAD+) beta	2.41	0.0219	2.19	0.0290
NM_004135	**IDH3G**	Isocitrate dehydrogenase 3 (NAD+) gamma	2.14	0.0262	2.29	0.0122
NM_005917	**MDH1**	Malate dehydrogenase 1, NAD (soluble)	9.51	0.0056	9.25	0.0039
BC001917	**MDH2**	Malate dehydrogenase 2, NAD (mitochondrial)	2.77	0.0739	3.48	0.0221
NM_004168	**SDHA**	Succinate dehydrogenase complex, A, flavoprotein	4.17	0.0108	4.03	0.0087
NM_003000	**SDHB**	Succinate dehydrogenase complex, B, iron sulfur	5.98	0.0120	6.68	0.0056
AF080579	**SDHC**	Succinate dehydrogenase complex, C, membrane protein	14.12	0.0003	11.63	0.0003
AL050226	**SUCLG2**	Succinate-CoA ligase, GDP-forming, beta	4.23	0.0108	2.83	0.0442
***Glycolysis***						
NM_000034	**ALDOA**	Aldolase A, fructose-bisphosphate	2.03	0.0136	3.05	0.0004
AK026411	**ALDOB**	Aldolase B, fructose-bisphosphate	14.83	0.0002	6.02	0.0027
AK026525	**GAPDH**	Glyceraldehyde-3-phosphate dehydrogenase	6.59	0.0034	4.89	0.0066
M69051	**GCK**	Glucokinase (hexokinase 4)	3.92	0.0010	1.01	0.9980
S81916	**PGK1**	Phosphoglycerate kinase 1	23.59	0.0009	23.43	0.0005
NM_002633	**PGM1**	Phosphoglucomutase 1	6.28	0.0038	5.03	0.0058
***Glycogen metabolism***						
S70004	**GYS2**	Glycogen synthase 2, liver	9.65	0.0004	5.54	0.0020
NM_002863	**PYGL**	Phosphorylase glycogen, liver	4.96	0.0120	3.14	0.0485
NM_002633	**PGM1**	Phosphoglucomutase 1	6.28	0.0038	5.03	0.0058
***Gluconeogenesis***						
D26054	**FBP1**	Fructose-1,6-bisphosphatase 1	4.08	0.0061	1.59	0.1559
BC020700	**G6PC**	Glucose-6-phosphatase, catalytic	12.13	0.0034	5.17	0.0265
***Others***						
NM_000284	**PDHA1**	Pyruvate dehydrogenase (lipoamide) alpha 1	3.48	0.0769	4.53	0.0249

^a ^Mean fold changes were calculated using a division of raw expression values between experimental sample and uninfected control.

^b ^All samples were analyzed separately. Changes in gene expression with a cutoff of 2.0-fold increased or decreased expression was used and, the *p*-value was calculated by Student's t-test. Data are displayed only where the, *p *≤ 0.05. This *p*-value was used as a measure of the magnitude of the difference between groups and to determine significance of the modulation. The modulated genes in the pathway are listed in alphabetic order.

**Table 4:** Expression of genes involved with fatty acid oxidation in LCMV-WE infected macaque liver

			**Pre-viremic**		**Viremic**	
				
**GenBank Accession no.**	**Symbol**	**Gene description**	**Fold change^a^**	***p*^b^**	**Fold change^a^**	***p*^b^**
***Mitochondrial {beta}-oxidation***						
BE897866	**ACADSB**	Acyl-CoA dehydrogenase, short/branched chain	1.77	0.6240	**-2.93**	0.0190
NM_004453	**ETFDH**	Electron-transferring flavoprotein dehydrogenase	3.10	0.0019	1.49	0.1412
NM_004092	**ECHS1**	Enoyl CoA hydratase, short chain, 1, mitochondrial	7.06	0.0110	4.79	0.0260
U04627	**HADHA**	Hydroxyacyl-CoA dehydrogenase/3-ketoacyl-CoA thiolase/enoyl-CoA hydratase (trifunctional protein), alpha	3.43	0.0180	3.75	0.0086
***Microsomal oxidation***						
AF182275	**CYP2A6**	Cytochrome P450, family 2, subfamily A, polypeptide 6	17.88	0.0008	7.21	0.0009
X06399	**CYP2B6**	Cytochrome P450, family 2, subfamily B, polypeptide 6	3.16	0.0079	-1.02	0.9980
NM_030878	**CYP2C8**	Cytochrome P450, family 2, subfamily C, polypeptide 8	3.51	0.0020	2.35	0.0147
NM_000106	**CYP2D6**	Cytochrome P450, family 2, subfamily D, polypeptide 6	7.78	0.0065	2.66	0.1530
AF182276	**CYP2E1**	Cytochrome P450, family 2, subfamily E, polypeptide 1	4.06	0.0150	2.87	0.0459
NM_000777	**CYP3A5**	Cytochrome P450, family 3, subfamily A, polypeptide 5	7.57	0.0005	6.36	0.0005
AF315325	**CYP3A7**	Cytochrome P450, family 3, subfamily A, polypeptide 7	11.39	0.0006	5.28	0.0050
***Ketogenesis***						
BC000408	**ACAT2**	Acetyl-CoA acetyltransferase 2 (acetoacetyl CoA thiolase)	6.10	0.0065	4.78	0.0107
BG035985	**HMGCS1**	3-hydroxy-3-methylglutaryl-CoA synthase 1 (soluble)	3.10	0.0198	4.69	0.0467
NM_005518	**HMGCS2**	3-hydroxy-3-methylglutaryl-CoA synthase 2 (mitochondrial)	5.16	0.0135	2.91	0.0748
BF224073	**TCP1**	T-complex 1	3.43	0.0332	2.50	0.0977
***Peroxisomal oxidation***						
NM_003500	**ACOX2**	Acyl-CoA oxidase 2, branched chain	6.63	0.0019	3.12	0.0267
NM_004092	**ECHS1**	Enoyl CoA hydratase, short chain, 1, mitochondrial	7.06	0.0110	4.79	0.0260
U04627	**HADHA**	Hydroxyacyl-CoA dehydrogenase/3-ketoacyl-CoA thiolase/enoyl-CoA hydratase (trifunctional protein), alpha	3.43	0.0180	3.75	0.0086
NM_005525	**HSD11B1**	Hydroxysteroid (11-beta) dehydrogenase 1	2.80	0.0325	2.20	0.0874
AL031228	**HSD17B8**	Hydroxysteroid (17-beta) dehydrogenase 8	2.80	0.0258	2.20	0.0620
***Other peroxisomal proteins***						
AU147084	**CAT**	Catalase	8.63	0.0028	5.78	0.0069
***Others***						
NM_000072	**CD36**	CD36 molecule (thrombospondin receptor)	48.18	0.0001	16.11	0.0008

^a ^Mean fold changes were calculated using a division of raw expression values between experimental sample and uninfected control.

^b ^All samples were analyzed separately. Changes in gene expression with a cutoff of 2.0-fold increased or decreased expression was used and, the *p*-value was calculated by Student's t-test. Data are displayed only where the, *p *≤ 0.05. This *p*-value was used as a measure of the magnitude of the difference between groups and to determine significance of the modulation. The modulated genes in the pathway are listed in alphabetic order.

**Table 5:** Expression of genes involved with fatty acid, cholesterol and amino acid metabolism in LCMV-WE infected macaque liver.

			**Pre-viremic**		**Viremic**	
				
**GenBank Accession no.**	**Symbol**	**Gene description**	**Fold change^a^**	***p*^b^**	**Fold change^a^**	***p*^b^**
***Fatty acid metabolism***						
AI057637	**ACACB**	Acetyl-Coenzyme A carboxylase beta	**-3.58**	0.0103	**-4.38**	0.0027
NM_001096	**ACLY**	ATP citrate lyase	2.14	0.0809	3.58	0.0040
AB032261	**SCD**	Stearoyl-CoA desaturase (delta-9-desaturase)	2.06	0.0448	4.69	0.0420
***Cholesterol metabolism***						
BC000408	**ACAT2**	Acetyl-CoA acetyltransferase 2 (acetoacetyl CoA thiolase)	6.10	0.0065	4.78	0.0107
U40053	**CYP51A1**	Cytochrome P450, family 51, subfamily A, polypeptide 1	3.68	0.0622	12.04	0.0010
NM_014762	**DHCR24**	24-dehydrocholesterol reductase	5.28	0.0006	3.66	0.0021
BC003573	**FDFT1**	Farnesyl-diphosphate farnesyltransferase 1	5.70	0.0195	6.02	0.0011
NM_002004	**FDPS**	Farnesyl diphosphate synthase	3.63	0.0218	5.03	0.0040
BG035985	**HMGCS1**	3-hydroxy-3-methylglutaryl-CoA synthase 1 (soluble)	3.01	0.0198	4.69	0.0467
NM_005336	**HDLBP**	High density lipoprotein binding protein (vigilin)	4.96	0.0007	3.14	0.0042
AF478446	**NR1H4**	Nuclear receptor subfamily 1, group H, member 4	11.31	0.0003	10.48	0.0002
BF530535	**SPCS2**	Signal peptidase complex subunit 2	10.34	0.0009	8.28	0.0011
***Amino acid metabolism***						
NM_000687	**AHCY**	S-adenosylhomocysteine hydrolase	7.46	0.0005	4.03	0.0048
AF110329	**GLS2**	Glutaminase 2	30.06	0.0039	11.31	0.0201
NM_002080	**GOT2**	Glutamic-oxaloacetic transaminase 2, mitochondrial (aspartate aminotransferase 2)	4.53	0.0036	3.12	0.0140
NM_000531	**OTC**	Ornithine carbamoyltransferase	4.47	0.0182	2.58	0.1090

^a ^Mean fold changes were calculated using a division of raw expression values between experimental sample and uninfected control.

^b ^All samples were analyzed separately. Changes in gene expression with a cutoff of 2.0-fold increased or decreased expression was used and, the *p*-value was calculated by Student's t-test. Data are displayed only where the, *p *≤ 0.05. This *p*-value was used as a measure of the magnitude of the difference between groups and to determine significance of the modulation. The modulated genes in the pathway are listed in alphabetic order.

**Table 6:** Expression of various lipase genes and genes involved with lipase activity in LCMV-WE infected macaque liver.

			**Pre-viremic**		**Viremic**	
				
**GenBank Accession no**	**Symbol**	**Gene description**	**Fold change^a^**	***p*^b^**	**Fold change^a^**	***p*^b^**
NM_000483	**APOC2**	Apolipoprotein C-II	5.50	0.0003	3.53	0.0019
AK023348	**GRN**	Granulin	1.84	0.0448	3.20	0.0007
NM_006854	**KDELR2**	KDEL (Lys-Asp-Glu-Leu) endoplasmic reticulum protein retention receptor 2	7.46	0.0056	7.36	0.0038
NM_000235	**LIPA**	Lipase A, lysosomal acid, cholesterol esterase	2.17	0.1350	4.08	0.0059
NM_006033	**LIPG**	Lipase, endothelial	2.20	0.4480	2.55	0.2950
AF077198	**LYPLA1**	Lysophospholipase I	7.16	0.0006	3.81	0.0008
BC006230	**MGLL**	Monoglyceride lipase	3.27	0.0090	2.73	0.0162
D83485	**PDIA3**	Protein disulfide isomerase family A, member 3	6.36	0.0028	7.46	0.0010
AL542253	**PLA1A**	Phospholipase A1 member A	1.37	0.0035	3.01	0.0124
NM_000300	**PLA2G2A**	Phospholipase A2, group IIA (platelets)	2.19	0.7700	26.17	0.0218
AF145020	**PLAA**	Phospholipase A2-activating protein	3.51	0.0026	2.69	0.0077
NM_002937	**RNASE4**	Ribonuclease, RNase A family, 4	45.25	0.00008	25.81	0.0001

^a ^Mean fold changes were calculated using a division of raw expression values between experimental sample and uninfected control.

^b ^All samples were analyzed separately. Changes in gene expression with a cutoff of 2.0-fold increased or decreased expression was used and, the *p*-value was calculated by Student's t-test. Data are displayed only where the, *p *≤ 0.05. This *p*-value was used as a measure of the magnitude of the difference between groups and to determine significance of the modulation. The modulated genes in the pathway are listed in alphabetic order.

The largest number of genes with altered expression contribute to lipid and glucose metabolism, and hence to the generation of energy for the organism. Key regulators of cholesterol synthesis, the mRNA for 3-hydroxy-3-methylglutaryl-CoA synthase 1 and 2 (HMGCS1, HMGCS2), were up-regulated during LCMV infection. Nevertheless, the liver-specific transporter gene APOAV was down-regulated (Additional File 8) suggesting that transport of triglycerides and cholesterol (via HDL/LDL) would be decreased in LCMV-infected liver. This is corroborated by lowered serum triglycerides during the pre-viremic and viremic stages (averaging 28 mg/dL when the norm is 50–82 mg/dL; Table 1). However blood chemistry showed that cholesterol levels were not affected by LCMV infection, so several transcriptional changes were not obviously related to effects on downstream metabolic products. A possibility is that increased energy-generating activity was defeated by poor transport, or that post-transcriptional regulation over-shadowed the rise in transcript levels.

### Effect of LCMV infection on glucose metabolism

**Figure 3:**
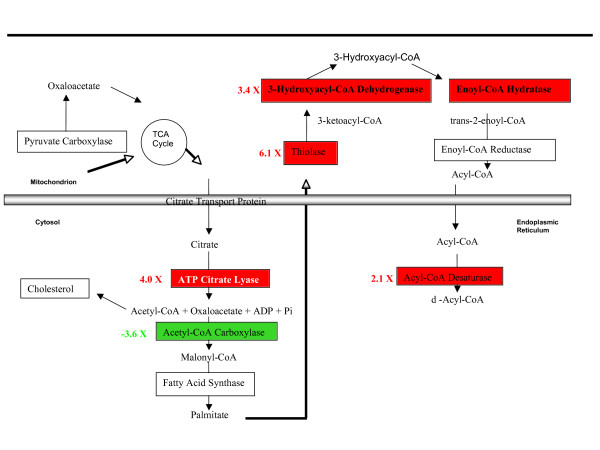
**Subcellular localization and metabolic pathways of significantly-modulated genes in the fatty acid synthesis pathway during the pre-viremic stage of LCMV-WE infection in macaque liver tissues**. Red or green color corresponds to up- or down-regulation of gene expression, respectively. ATP citrate lyase is encoded by ACLY (Tables 3 and 5), Acetyl-CoA carboxylase is encoded by ACACB (Table 4), thiolase is encoded by ACAT2 (Tables 3 and 4), 3-hydroxyacyl-CoA dehydrogenase is encoded by HADHA (Table 4), enoyl-CoA hydratase is encoded by ECHS1 (Table 4) and acyl-CoA desaturase is encoded by SCD (Table 5).

Monkeys undergoing virulent infection had normal blood glucose levels (except at the terminal stage; Table 1) up-regulated numerous genes involved in carbohydrate metabolism (Additional File 9, tables 3, 4, 5 and 6). During the pre-viremic stage of infection several genes were up-regulated for enzymes involved in gluconeogenesis, i.e. the breakdown of fatty acids, amino acids and pyruvate to make glucose (Figure 2, Tables  3, 4, 5, and 6). For example, glucose-6-phosphatase (G6Pase) was up-regulated in both pre-viremic and viremic stages, and fructose 1,6-bis phosphatase (FBP1) was four-fold up-regulated only at the pre-viremic stage (Table 3). The increased gluconeogenesis during LCMV infection resembled that seen during starvation [23] or cachexia [24], however the infection also up-regulated mRNA encoding glycolytic enzymes (PGK1, GCK, GAPDH and ALDOB; Table 3) that are usually down-regulated during a fast [25]. Simultaneous up-regulation of glycolytic and gluconeogenic enzymes in infected animals could mean that sugar metabolism is in a futile cycle and that the majority of energy production during LCMV infection comes from beta-oxidation of fatty acids (Tables 3, 4 and 5, Figure 3).

LCMV-infected liver up-regulated expression of glycogen synthase 2 (GYS2) in both pre-viremic and viremic stages of infection (Table 3) suggesting the possibility that glucose storage was enhanced. Glycogen in the LCMV-infected liver could also be derived from hydrolysis of triglycerides in fat cells since several lipases and beta-oxidation enzymes were up-regulated (Table 7) and triglycerides in blood were unusually low (Table 1).

**Table 7:** Expression of genes involved with complement and coagulation cascades in LCMV-WE-infected macaque liver

			**Pre-viremic**		**Viremic**	
				
**GenBank Accession no.**	**Symbol**	**Gene description**	**Fold change^a^**	***p*^b^**	**Fold change^a^**	***p*^b^**
NM_000014	**A2M**	Alpha-2-macroglobulin	3.31	0.0219	2.71	0.0417
NM_000491	**C1QB**	Complement component 1, q subcomponent, B chain	3.65	0.1140	12.2	0.0029
AI184968	**C1QC**	Complement component 1, q subcomponent, C chain	1.47	0.5402	3.27	0.0140
AL573058	**C1R**	Complement component 1, r subcomponent	2.20	0.0287	2.73	0.0054
BC007010	**C1S**	Complement component 1, s subcomponent	53.44	0.0006	50.21	0.0004
NM_000592	**C4A/C4B**	Complement component 4A (Rodgers blood group)///complement component 4B (Childo blood group)	5.20	0.0002	6.32	0.0001
NM_000716	**C4BPB**	Complement component 4 binding protein, beta	3.22	0.0401	5.65	0.0032
NM_001735	**C5**	Complement component 5	8.75	0.0015	7.83	0.0013
J05064	**C6**	Complement component 6	3.24	0.0332	2.63	0.0624
NM_000587	**C7**	Complement component 7	2.34	0.1850	4.28	0.0152
M17263	**C8G**	Complement component 8, gamma polypeptide	4.72	0.0051	4.56	0.0038
AL570661	**CD46**	CD46 molecule, complement regulatory protein	4.72	0.0015	3.24	0.0060
X04697	**CFH**	Complement factor H	2.90	0.0124	2.86	0.0091
NM_000506	**F2**	Coagulation factor II (thrombin)	2.11	0.0329	1.65	0.1360
AA910306	**F5**	Coagulation factor V (proaccelerin, labile factor)	3.16	0.0218	2.47	0.0071
NM_000504	**F10**	Coagulation factor X	2.42	0.0070	1.85	0.0513
NM_000128	**F11**	Coagulation factor XI (plasma thromboplastin antecedent)	**-2.28**	0.0150	**-2.75**	0.0029
NM_021871	**FGA**	Fibrinogen alpha chain	3.86	0.0035	3.48	0.0039
BG545288	**FGB**	Fibrinogen beta chain	49.86	0.0001	45.56	0.0001
NM_000509	**FGG**	Fibrinogen gamma chain	2.67	0.0355	2.56	0.0325
M74220	**LPA/PLG**	Lipoprotein, Lp(a)///plasminogen	4.69	0.0256	3.81	0.0396
NM_000892	**KLKB1**	Kallikrein B, plasma (Fletcher factor) 1	**-1.70**	0.1730	**-2.32**	0.0207
AI274095	**MASP1**	Mannan-binding lectin serine peptidase 1 (C4/C2 activating component of Ra-reactive factor)	3.07	0.0004	2.78	0.0005
AB008047	**MASP2**	Mannan-binding lectin serine peptidase 2	4.75	0.0144	3.16	0.0489
NM_000242	**MBL2**	Mannose-binding lectin (protein C) 2, soluble (opsonic defect)	3.18	0.0162	1.37	0.5910
BE880828	**MCFD2**	Multiple coagulation factor deficiency 2	11.71	0.0004	14.72	0.0001
M74220	**PLG**	Plasminogen	3.86	0.0059	3.60	0.0054
NM_000295	**SERPINA1**	Serpin peptidase inhibitor, clade A (alpha-1 antiproteinase, antitrypsin), 1	8.20	0.0003	7.21	0.0004
BC022309	**SERPINC1**	Serpin peptidase inhibitor, clade C (antithrombin), member 1	30.65	0.0005	19.29	0.0009
NM_000062	**SERPING1**	Serpin peptidase inhibitor, clade G (C1 inhibitor), member 1	2.77	0.0336	3.07	0.0296
BF511231	**TFPI**	Tissue factor pathway inhibitor (lipoprotein-associated coagulation inhibitor)	2.05	0.0312	1.45	0.2590

^a ^Mean fold changes were calculated using a division of raw expression values between experimental sample and uninfected control.

^b ^All samples were analyzed separately. Changes in gene expression with a cutoff of 2.0-fold increased or decreased expression was used and, the *p*-value was calculated by Student's t-test. Data are displayed only where the, *p *≤ 0.05. This *p*-value was used as a measure of the magnitude of the difference between groups and to determine significance of the modulation. The modulated genes in the pathway are listed in alphabetic order.

### Effect of virulent infection on lipogenic pathway (fatty acid metabolism)

Mobilization of fat (lipolysis) is favored under conditions of increased energy need, such as exercise, fasting, hypothermia [23], or cachexia associated with cancer and AIDS [24]. When triglycerides in adipose tissue are broken down by lipase, then the fatty acids and glycerol are released to the bloodstream. Genes involved in fatty acid transport, albumin (ALB), myosin (MYO1B), and the thrombospondin receptor (CD36), were up-regulated during infection (Figure 2). In the mitochondria, fatty acids undergo β-oxidation into two-carbon acetyl groups attached to CoA [23]. During LCMV infection, genes for fatty acid metabolic enzymes were strongly up-regulated: thiolase (ACAT2), hydroxyacyl-CoA dehydrogenase (HADHA), enoyl-CoA hydratase (ECHS1), and stearol-CoA desaturase (SCD) (Tables 4 and 5). The only down-regulated gene in the fatty acid metabolic group was acetyl-CoA carboxylase (ACACB), a major regulator of fatty acid synthesis [23]. This would predict that free fatty acids arose more frequently by β-oxidation than by fatty acid synthesis via ACACB (Figure 3; Table 5). In support of that prediction, several cytochrome P450 isoforms (CYP2A, -3A, -2B) were up-regulated that are known to break down steroids and fats during the course of LCMV-infection (Table 4) [26,27]. The down-regulation of ACACB and the up-regulation of CYP26A1 have been validated by quantitative PCR (Table 2).

Free fatty acids (FFA) stimulate key gluconeogenic enzymes [28,29]. For example the enzyme encoded by PCK is up-regulated by FFA and peroxisome proliferator-activated receptor (encoded by PPAR) in hepatoma cells [30]. However both PPAR and PCK are more up-regulated in the mild (non-diseased) infections than in the virulent infections (Additional File 8), contributing to the picture that these changes in intermediary metabolism are a protective host response that is stronger in the non-diseased cases.

The aldehyde dehydrogenases clear the blood of toxic aldehydes by generating acetate, which can be converted to acetyl CoA. Several aldehyde dehydrogenases are up-regulated during infection, especially ALDH6A1, a liver mitochondrial enzyme that catalyzes the breakdown of malonate to propionyl – and acetyl-CoA, (up-regulated 37- and 22-fold in pre-viremic and viremic stages, Additional File 8). Levels of acetyl-CoA are central to the balance between carbohydrate and fat metabolism, so in the LCMV-infected liver, it appears that the product of ALDH6A1 could drive acetyl CoA into the cholesterol or citric acid cycles to generate energy but not into fatty acid synthesis due to down-regulated ACACB acting as a bottleneck (Figure 3).

Ketone-body formation (ketogenesis) occurs during high rates of fatty acid oxidation through generation of large amounts of acetyl-CoA in liver [23]. Ketone bodies, consisting of acetoacetate, acetone and β-hydroxybutyrate, serve as an alternative source of energy at low glucose levels. Ketogenesis was likely higher in LCMV-WE infected rhesus macaques than in the mildly-infected macaques since fatty acid synthesis via acetyl-coenzyme A carboxylase beta (ACACB) was more down-regulated in the virulent infection (Figure 2; Table 5). Genes involved with mitochondrial fatty acid oxidation, cholesterol synthesis and ketogenesis (Table 3; Additional File 8), for example, the hydroxy-3-methylglutaryl-CoA synthases (HMGCS1 and 2) were induced by LCMV infection. They condense acetyl-CoA and acetoacetyl-CoA to form HMG-CoA, an intermediate in the pathway for synthesis of ketone bodies, a likely source of energy during LCMV-WE infection (Table 5).

The expression of several lipase genes is strongly up-regulated during LCMV-WE infection, suggesting a relationship between up-regulation of beta-oxidation enzymes and lipolysis in the infected livers (Table 6). In general, we found that genes involved in degradation of lipids and fatty acids were up-regulated while a key gene involved in fatty acid synthesis was down-regulated.

### Effect of LCMV infection on gene expression related to coagulation and complement cascade pathways

The complement and coagulation cascades interact and modulate each other; both are affected during LCMV infection. Although we do not present coagulation data here, platelet defects and a mild thrombocytopenia were documented during severe Lassa fever [11,12], and macaques with virulent LCMV disease showed a marked thrombocytopenia [4]. The transcriptome data (Table 7) show over-expression of complement component 1 (C1S), an anti-thrombin peptide (SERPINC1), and fibrinogen beta chain (FGB) during virulent infection. Over-expression of C1S and FGB have been validated by quantitative PCR (Table 2). These 3 genes alone would predict problems with coagulation: high C1S should drive the classical pathway of the complement cascade towards more phagocyte recruitment and cell lysis, high serpin should inhibit the intrinsic pathway of coagulation, and high fibrinogen should overwhelm the fibrinolysis that occurs during clot formation [31]. Additionally, the multiple coagulation factor deficiency 2 (MCFD2) gene is up-regulated 11-fold in pre-viremic and 15-fold during the viremic phase and is important in the secretion of coagulation factors V and VIII [32]. In addition to these highly over-expressed genes, there is modest up-regulation of coagulation factors II, V, and X which should promote coagulation and has been associated with consumptive coagulopathy and disseminated intravascular coagulation (DIC); although DIC has not been associated with Lassa [13] as it has with other hemorrhagic virus infections like Ebola fever [33]. We also observed down-modulation of a gene encoding kallikrein (KLKB1) that dilates blood vessels and should prevent vessel leakage.

### Gene expression profiling that discriminates between virulent and non-virulent virus infection in LCMV-infected liver

**Figure 4:**
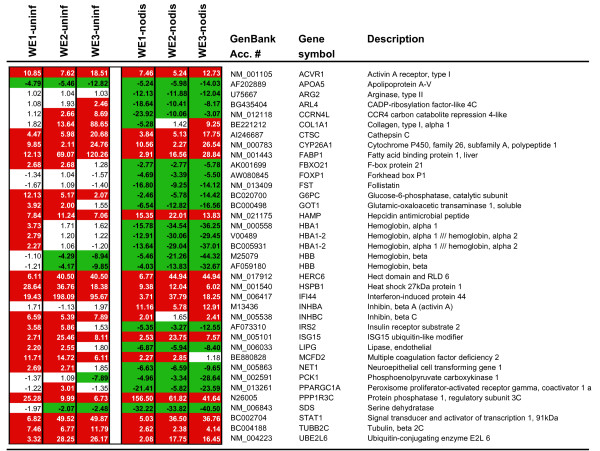
**Listing of the most differentially-modulated genes in liver with respect to infected-but not-diseased samples**. There are clear differences between infected and uninfected samples, but less frequently, there are also differences between virulently-infected and mildly-infected samples and the most prominent genes in this latter category are highlighted in this figure. WE1 vs uninf (or vs nodisease) refers to pre-viremic samples (day 1–3) and WE2 vs uninf (or vs nodisease) refers to viremic samples (day 4–7). N-fold refers to fold up- or down-regulation of each gene expressed in a virulently-infected liver in relation to expression in an uninfected or non-diseased liver. Gene accession number, gene symbol and description of each gene are shown. Red or green color corresponds to up- or down-regulation of gene expression, respectively. This figure is a subset of Additional File 8.

Genes that discriminate between virulent and non-virulent infection, especially if they do so during the pre-viremic stage, have the greatest potential as prognostic biomarkers for severe arenaviral disease. Virulent and non-virulent liver transcriptomes were compared by pooling results from nine animals (LCMV-WE-infected) to compare with six samples from animals that were LCMV-infected but not diseased (two animals infected with LCMV-ARM and four infected with both LCMV-WE and LCMV-ARM that did not develop disease). In our study, the transcriptome of non-diseased animals closely resembled the transcriptome of animals infected with non-virulent LCMV-ARM (see clustering in Additional File 1). Liver gene expression profiles showed significant differences between virulent and non-virulent infections (Figure 4). We determined that approximately 244 genes significantly discriminated non-virulent and virulent LCMV infections, and of these, approximately 30 did so during the pre-viremic stage of infection (Figure 4 and Additional File 8). Notable are large drops in expression of hemoglobin alpha and beta genes, despite up-regulation of transferrin (TF) the product of which promotes iron absorption and hemoglobin production [34]. Down-regulation of hemoglobin is concomitant with up-regulation of IL-6-inducible hepcidin (HAMP), an iron regulatory hormone known to block iron export and uptake [35]. Down-regulation of hemoglobin mRNA may also be related to a block in erythropoiesis caused by interferon [36]; since interferon levels are higher in virulent than in benign infection of primates [3]. The down-regulation of hemoglobin genes has been validated by quantitative PCR (Table 2).

As mentioned earlier, LCMV infection is predicted to promote significant amino acid breakdown by gluconeogenesis. Three amino acid catabolic enzymes, arginase (ARG2), serine dehydratase (SDS), and glutamic-oxaloacetic transaminase (GOT1), were not altered in their expression when comparing infected versus uninfected samples, yet there was a significant down-regulation of these genes when comparing LCMV-WE-infected to LCMV-non-diseased samples (Figure 4). This means that transcripts from mildly-infected samples are up-regulated with respect to transcripts from uninfected liver. The up-regulation of these samples in mild infections has been validated by quantitative PCR (Table 2). A similar pattern has been observed for peroxisome proliferators-activated receptor gamma (PPARGC1A), hemoglobin α1/α2 (HBA 1–2) and follistatin (FST) (Figure 4), and has been validated by quantitative PCR (Table 2). It is likely that increased expression of these genes protects animals with mild infections from a more severe disease.

PPP1R3C is the most dramatically up-regulated transcript in virulently-infected livers with respect to mildly-infected liver samples (156-fold in the pre-viremic stage), and this up-regulation has been validated by quantitative PCR (Table 2). PPP1R3C encodes an inhibitory subunit of protein phosphatase and is known to bind liver glycogen in response to high levels of insulin. It belonging to a family of pro-inflammatory genes with short-lived-mRNA controlled by AU-rich elements in their 3'UTR [37]. Expression of PPP1R3C has been associated with encephalopathy [38].

Additional notable differences between virulent and mild infections are large increases in expression of genes for activin receptor (ACVR1) and inhibin (INHBA, INHBC), along with a decrease in expression of follistatin (FST); all of which belong to the transforming growth factor beta (TGF-β) gene family. Expression of these TGF-β family genes was validated by real-time quantitative PCR (Table 2). Though the gene for TGF-β was not differentially expressed during LCMV infection, the protein was detectable in plasma during the viremic stage [3]. Follistatin is known to bind and inactivate inhibin, and the down-regulation of FST is related to the rise in INHB and ACVR1 expression [39]. The inhibins act as negative regulators of B cell development [40], which may contribute to LCMV-mediated suppression of anti-viral immune responses.

## Discussion

The LCMV-infected monkey model for Lassa fever was investigated with the goal of finding transcriptome changes that could eventually be used as prognostic biomarkers to discriminate virulent and mild infections. Transcriptome analysis of rhesus macaque liver showed that both mild and virulent LCMV infections had tremendous effects on glucose, amino acid and fatty acid metabolism, and on the complement and coagulation cascades. As with our previous transcriptome analysis using blood [3], prominent expression of inflammatory-response genes was also seen in liver.

A transcriptome study using the HuH-7 liver cell line [41] showed that Lassa infection up-regulated only laminin-a and ribosomal protein L28 with respect to gene expression in uninfected cells. It is frequently observed that studies of single cell types will show few differences in comparison to studies of complex tissues. Although our study did not see changes in expression of the laminin-a gene, several ribosomal protein genes were up-regulated in LCMV-infected liver: approximately 30 were moderately up-regulated, and 20 were highly up-regulated, with L28 being the most up-regulated of all (Additional File 8). None of the ribosomal gene expression changes observed in LCMV-infected liver differed significantly when comparing the virulent and mild infections. It is possible that L28 is a co-factor of arenavirus replication, since replicating LCMV is associated with ribosomal protein complexes [42-44].

Other liver transcriptome studies have identified major molecular events associated with viral infection, with higher levels of interferon-responsive genes being a common theme. We observed significant up-regulation of ISG15, IFIT1, and STAT1 in LCMV-WE-infected (diseased) monkey liver with respect to non-diseased liver, and similar increased expression was seen in human liver infected with hepatitis C virus [45]. As noted in a recent paper on the Ebola virus transcriptome [46], the majority of cells profiled are uninfected bystanders and are responding to plasma interferon despite the interferon-antagonistic activity of viral genes within infected cells [47]. In two liver transcriptome studies of human beings infected with hepatitis C virus, MHC class I and II genes were up-regulated in infected versus uninfected liver [45,48]; in our studies, several MHC genes are up-regulated in liver, with concomitant down-regulation in PBMC [3], possibly due to migration of activated monocytes from the circulation to infected sites. A noteable difference between the hepatitis C liver transcriptome and the LCMV-liver transcriptome is that Serpin D1 and C genes are down-regulated by hepatitis C, whereas Serpin C is 30-fold up-regulated in the LCMV liver (Figure 2) and would be expected to inhibit the intrinsic coagulation pathway.

Two crucial liver functions are glycogen breakdown and gluconeogenesis from non-sugars like fatty acids, pyruvate, lactate, and amino acids, with both functions beginning a few hours after the onset of fasting. Elevated rates of fat breakdown (lipolysis) increase the release of free fatty acids that simultaneously stimulate gluconeogenesis and inhibit glycogenolysis [49,50]. The oxidation of fatty acids by liver mitochondria leads to the generation of ketone bodies, which appear in the blood in inverse proportion to glucose and provide an important alternative fuel source [51,52]. In LCMV-infected monkey liver there was a significant increase in transcripts promoting gluconeogenesis in both stages of infection and a decrease in transcripts promoting glycogenolysis (e. g. G6PC and FBP1) in the early stage of infection. It is likely that gluconeogenesis was driven by hydrolysis of triglycerides since transcripts for β-oxidation enzymes and lipases were up-regulated in LCMV-infected livers (Table 6). Glycolysis and gluconeogenesis constituted a "futile cycle" that would be predicted to waste energy during LCMV infection (Table 2). During the early stage of infection the macaque liver increased the usage of fatty acids and ketone bodies, and later in the infection, the liver functioned in a glucose-producing role, using amino acids and fatty acids as an energy source.

Other viral infections are also inter-twined with glucose, amino acid and fatty-acid metabolism. The transcription of hepatitis B virus, a DNA virus, requires a key regulatory enzyme of gluconeogenesis, peroxisome proliferator-activated receptor γ C1 (PPARGC1[53]. This enzyme acts through nuclear receptors (the glucocorticoid receptor, the forkhead transcription factor, and the nuclear receptor 4a) to stimulate transcription of hepatitis B. PPARGC1 is known to be up-regulated by fasting, cold temperature or stress [53], and, remarkably, it is significantly up-regulated in the mild LCMV infection but not in the virulent infection; this has been validated by quantitative PCR (Table 2). Another glycolytic enzyme, phosphoglycerate kinase (PGK), stimulates Sendai virus transcription through its interaction with tubulin in the initiation complex [54]. Vero cells infected with the alphavirus, Mayaro, have altered glucose metabolism and increased glucose consumption [55], but it is unclear whether this is directly related to virus replication. The key glycolytic enzyme, glyceraldeyde 3-phosphate dehydrognase (GAPDH) is involved in the life cycle of parainfluenza virus type 3 (HPIV3) [56]. Interestingly, the genes for both PGK and GAPDH were strongly up-regulated in both mild and virulent LCMV infections. Hepatitis C virus (HCV) infection also affects hepatic glucose metabolism. HCV down-regulates insulin-receptor substrate genes (IRS1 and IRS2) through up-regulation of suppressor of cytokine signaling (SOCS) [57]. Similarly in the LCMV-infected primate, IRS1 expression is decreased and expression of IRS2 is moderate, but SOCS2 is up-regulated (7.2-fold) at the pre-viremic stage of infection (Additional File 8).

The acute LCMV disease has characteristics of starvation that resemble both cachexia (TNF-α or IL-6-mediated wasting) and anorexia (appetite loss). High levels of IL-6 were detected in LCMV-WE-infected liver [6]. IL-6 is known to stimulate protein catabolism in order to maintain glucose levels via gluconeogenesis [24], whereas anorexia, is largely driven by IL-1β [58] and is known to mobilize fat stores in lieu of protein stores [24]. Here, though we failed to detect increases in IL-1β, we observed increased expression of both lipid-oxidation and protein catabolism genes. We also observed an early decrease in blood triglycerides (Table 1) that corroborates our transcriptome result of suppressed ACACB. The low triglycerides we observe also contrast with the profile of bacterial sepsis in which high liver IL-6 coincides with high triglycerides [59]. Although high IL-6 is detected by day 7 [6] high circulating triglycerides are not seen until day 11 or 12 in the LCMV-WE-infected macaques (Table 1).

It is difficult to relate changes in intermediary metabolism of LCMV-infected liver to a lethal disease that resembles Lassa fever. Although the liver transcriptome may resemble that of a starving organism, and such a profile is supported by the dehydration and appetite-loss observed in diseased monkeys, it is known that primates can survive weeks of starvation [51]; so it is unlikely that starvation alone explains such a rapid disease. Since most of the transcriptome changes that affected intermediary metabolism were seen in both the diseased and non-diseased animals it is unlikely that those changes were the primary cause of disease. Importantly, several genes involved in intermediary metabolism (ARG2, GOT1, G6PC, IRS2, LIPG, PCK1, SDS, PPARGC1) were considerably more up-regulated in the mild than in the fatal infections (Figure 4) leading to the possibility that the altered energy metabolism is primarily a self-preserving rather than a pathogenic activity.

Some of the differences between virulently – and mildly-infected liver could be ascribed to the over-expression of interferon-response genes (e.g. IFI44, Figure 4). A large drop in hemoglobin gene expression in virulent infections was likely due to interferon inhibition of erythropoiesis [36]. Paradoxically, circulating levels of red blood cells and hemoglobin were unaffected by LCMV-WE infection (Table 1), but events in the liver may precede what is detectable in the circulation. A recent paper describes lethal hemorrhagic anemia in LCMV-infected mice that did not occur in IFN-α/β knockout mice [60]. In the murine model, both LCMV-WE and LCMV-ARM caused platelet dysfunction and life-threatening anemia. The murine studies used much higher doses of virus than we used for our primate pathogenesis studies, i. e. mice were given 10^6 ^pfu virus, whereas the monkeys were given 10^3 ^pfu. Also, the disease in mice is generally considered immunopathological, i.e. alleviated by immunosuppression, unlike the disease in guinea pigs and primates [61]. In the monkey model, only LCMV-WE replicates well in monkey liver whereas LCMV-ARM replicates poorly [4], and only LCMV-WE elicits detectable levels of interferon in plasma [8]. Nevertheless, it is quite reasonable that in the primate, IFN-α/β initiates platelet dysfunction, and it might be reasonable to treat the primate disease with platelet transfusions, as was done in the murine model.

## Conclusion

Microarray analysis identified several potential gene markers of LCMV-WE-associated liver disease. By investigating changes in gene expression during the early stages of disease we identified pathways most likely to be instigating the disease signs observed during viral hemorrhagic fever. Alterations in intermediary metabolism are more likely a sign of active resistance than of pathogenesis. Virus-mediated cytokine production could be responsible for curtailed erythropoiesis and platelet dysfunction. Changes in expression of genes in the coagulation cascade could be directly responsible for capillary leakage and thrombocytopenia in virulent LCMV infection.

## Competing interests

The authors declare that they have no competing interests.

## Authors' contributions

MD participated in study design, processed the liver samples, oversaw the workflow, participated in bioinformatics analyses and wrote the first drafts of the manuscript. ORC contributed to study design, and oversaw the bioinformatic analyses of the chip hybridizations. YZ performed much of the bioinformatics analyses and uploaded the data to the GEO database, JCZ contributed to study design and was responsible for animal sampling and sample organization. BS oversaw the workflow at the Virginia Bioinformatics Institute where ORC and YZ conducted analyses. MGL validated liver gene expression using real-time RT-PCR and contributed edits to the final manuscript. JB oversaw the animal model and contributed to study design. HD cared for the animals and oversaw the sampling. MS conceived of the study, participated in design and edited the final drafts of the manuscript. All authors read and approved the final manuscript.

## Supplementary Material

Additional File 1 **Cluster analysis of differentially-expressed genes**. Cluster analysis of differentially expressed genes representing 4482 probe sets from uninfected or LCMV-infected liver samples filtered by having p < 0.05 and at least 20 percent of present call. Each row represents the indicated gene. Each column corresponds to the experimental liver sample as listed at the top. Red indicates an up-regulation of gene expression and green indicates a down-regulation of expression in rhesus macaque liver. The origin of each sample is described in Table 1. Click here for file

Additional File 2 **Lists genes from the Venn diagram of Figure **1 i**n the form of 2751 probe sets**. "Pre" means they were expressed in the pre-viremic stage (day 1–3) in blood, but not in liver. Click here for file

Additional File 3 **Lists genes from the Venn diagram of Figure **1**in the form of 500 probe sets**. "Pre" means they were expressed in the pre-viremic stage (day 1–3) in both blood and liver. Click here for file

Additional File 4 **Lists genes from the Venn diagram of Figure **1**in the form of 2033 probe sets**. "Pre" means they were expressed in the pre-viremic stage (day 1–3) in liver, but not in blood. Click here for file

Additional File 5 **Lists genes from the Venn diagram of Figure **1** in the form of 3136 probe sets**. "Vir" means they were expressed in the viremic stage (day 4–7) in blood, but not in liver. Click here for file

Additional File 6 **Lists genes from the Venn diagram of Figure **1**in the form of 963 probe sets**. "Vir" means they were expressed in the viremic stage (day 4–7) in blood and liver. Click here for file

Additional File 7 **Lists genes from the Venn diagram of Figure **1**in the form of 1722 probe sets.** "Vir" means they were expressed in the viremic stage (day 4–7) in liver, but not in blood. Click here for file

Additional File 8 **List of differentially-expressed genes in LCMV-infected liver**. This list of human probe sets identified in hybridization of rhesus macaque liver cRNA includes those transcripts differentially expressed with respect to uninfected (uninf) control samples (n = 4484), or those transcripts differentially expressed with respect to samples from monkeys that were LCMV-infected but not diseased (nodis). These were identified as differentially expressed using LIMMA analysis. WE1 refers to 4 liver samples taken day 1 to day 3 after infection (pre-viremic stage). WE2 refers to 5 liver samples taken day 4 to day 7 after infection (viremic stage), and WE3 refers to 2 liver samples taken day 11 and day 12 after infection (terminal stage). WEx vs uninf refers to virulent samples being compared to data from 3 uninfected samples. WEx vs nodis refers to virulent samples being compared to data from 6 infected but not diseases samples. Samples are more fully described in Table 1 of the manuscript. Mean fold changes were calculated using a division of log (2)-normalized expression values between experimental sample and uninfected control as described in Materials & Methods. False discovery rate of the pair-wise comparisons were calculated using *p*-values from LIMMA. Significantly regulated genes were selected using a 2-fold cut-off and a false discovery rate of < 0.05. Click here for file

Additional File 9 **Pathway gene expression in LCMV-WE-infected macaque liver tissues.** Significantly affected pathway gene expression in LCMV-WE-infected macaque liver tissues. KEGG software was used to identify groupings of genes with pathways based on published data. False discovery rate of the pair-wise comparisons were calculated using *p*-values from LIMMA. Significantly regulated genes were selected using a 2-fold cut-off and a false discovery rate of < 0.05. Click here for file
