# Supplementary figures and images for: Gene expression in primate liver during viral hemorrhagic fever
Source: Virol J. 2009 Feb 12;6:20. doi: 10.1186/1743-422X-6-20 (PMC2657139; doi:10.1186/1743-422X-6-20)

## Slide 1
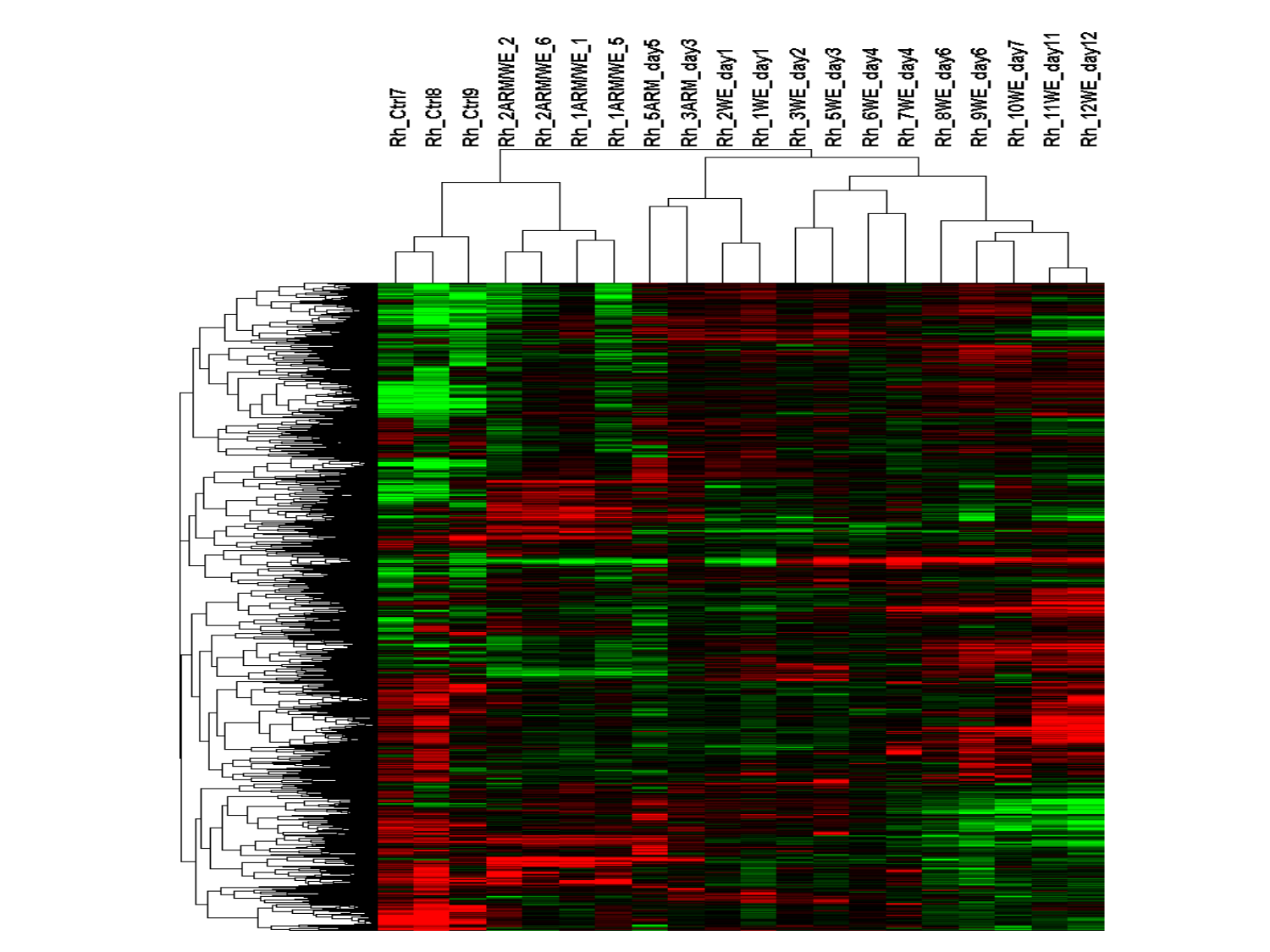

Supplement: Additional File 1 — Cluster analysis of differentially-expressed genes. Cluster analysis of differentially expressed genes representing 4482 probe sets from uninfected or LCMV-infected liver samples filtered by having p < 0.05 and at least 20 percent of present call. Each row represents the indicated gene. Each column corresponds to the experimental liver sample as listed at the top. Red indicates an up-regulation of gene expression and green indicates a down-regulation of expression in rhesus macaque liver. The origin of each sample is described in Table 1. [file 1743-422X-6-20-S1.ppt]
