# Supplementary material for: Gene expression in primate liver during viral hemorrhagic fever
Source: Virol J. 2009 Feb 12;6:20. doi: 10.1186/1743-422X-6-20 (PMC2657139; doi:10.1186/1743-422X-6-20)
Supplement: Additional File 9 — Pathway gene expression in LCMV-WE-infected macaque liver tissues. Significantly affected pathway gene expression in LCMV-WE-infected macaque liver tissues. KEGG software was used to identify groupings of genes with pathways based on published data. False discovery rate of the pair-wise comparisons were calculated using p-values from LIMMA. Significantly regulated genes were selected using a 2-fold cut-off and a false discovery rate of < 0.05. [file 1743-422X-6-20-S9.doc]

**Additional File 2, Table II: Modulation of pathway gene expression in LCMV-WE-infected macaque liver**

**GenBank Pre-viremic Viremic**

**Accession no. Symbol Gene description Fold Fold**

**change^a^ *p*^b^ change^a^ *p*^b^**

***Androgen and estrogen metabolism;***

AW168942 ARSB Arylsulfatase B 3.00 0.0010 3.13 0.0007

NM_005525 HSD11B1 Hydroxysteroid (11-beta) dehydrogenase 1 2.78 0.0325 2.15 0.0874

AL031228 HSD17B8 Hydroxysteroid (17-beta) dehydrogenase 8 2.78 0.0258 2.21 0.0620

NM_003167 SULT2A1 Sulfotransferase family, cytosolic, 2A,

(Dehydroepiandrosterone (DHEA)-preferring, member 1) 4.99 0.0030 3.86 0.0023

NM_019093 UGT1A1-10 UDP glucuronosyltransferase 1 family, polypeptide A1-10 20.96 0.0002 13.08 0.0004

NM_019076 UGT1A8-9 UDP glucuronosyltransferase 1 family, polypeptide A8-9 27.85 0.0003 8.28 0.0053

***Apoptosis***

NM_004208 AIFM1 Apoptosis-inducing factor, mitochondrion-associated, 1 3.22 0.0127 2.44 0.0382

NM_001166 BIRC2 Baculoviral IAP repeat-containing 2 3.53 0.0100 5.61 0.0008

AF327443 CAST Calpastatin 2.60 0.0018 2.96 0.0005

BC005299 CYCS Cytochrome c, somatic 3.03 0.0439

AU153366 IKBKB Inhibitor of kappa light polypeptide gene enhancer in

B-cells, kinase beta 3.48 0.0049 4.22 0.0012

AF074382 IKBKG Inhibitor of kappa light polypeptide gene enhancer

in B-cells, kinase gamma  **-2.09** 0.0011  **-2.02** 0.0010

AK026803 IL1R1 Interleukin 1 receptor, type I 6.10 0.0046 8.51 0.0010

AI078167 NFKBIA Nuclear factor of kappa light polypeptide gene enhancer

in B-cells inhibitor, alpha 2.32 0.0934 2.78 0.0313

AI679268 PIK3R1 Phosphoinositide-3-kinase, regulatory subunit 1 (p85 alpha) 3.83 0.0249 2.36 0.1320

BF740111 PIK3R4 Phosphoinositide-3-kinase, regulatory subunit 4, p150 2.71 0.0246 1.69 0.2230

AV682436 PIK3C2A Phosphoinositide-3-kinase, class 2, alpha polypeptide 2.60 0.0161 2.47 0.0150

AA130247 PRKACB Protein kinase, cAMP-dependent, catalytic, beta 2.53 0.0411 3.01 0.0124

NM_002734 PRKAR1A Protein kinase, cAMP-dependent, regulatory, type I, alpha 5.89 0.0128 4.40 0.0240

AK026351 PRKAR2A Protein kinase, cAMP-dependent, regulatory, type II, alpha 3.11 0.0127 1.37 0.5550

NM_003810 TNFSF10 Tumor necrosis factor (ligand) superfamily, member 10 8.00 0.0062 9.51 0.0024

BF508371 TNFRSF1A Tumor necrosis factor receptor superfamily, member 1A  **-4.78** 0.0008  **-4.46** 0.0007

***ATP Synthesis***

NM_001686 ATP5B ATP synthase, mitochondrial F1 complex, beta polypeptide 5.42 0.0125 6.27 0.0052

NM_005174 ATP5C1 ATP synthase, mitochondrial F1 complex, gamma polypeptide 1 3.68 0.0290 3.70 0.0212

NM_001687 ATP5D ATP synthase, mitochondrial F1 complex, delta subunit 2.39 0.0379 2.31 0.0361

NM_006886 ATP5E ATP synthase, mitochondrial F1 complex, epsilon subunit 2.58 0.0062 2.84 0.0021

NM_001689 ATP5G3 ATP synthase, mitochondrial F0 complex, subunit C3 (subunit 9) 6.10 0.0167 7.31 0.0066

AF061735 ATP5H ATP synthase, mitochondrial F0 complex, subunit D 23.91 0.0001 26.53 0.0000

NM_004889 ATP5J2 ATP synthase, mitochondrial F0 complex, subunit F2 3.53 0.0555 5.54 0.0085

NM_001685 ATP5J ATP synthase, mitochondrial F0 complex, subunit F6 2.75 0.0488 3.58 0.0110

AF070655 ATP5L ATP synthase, mitochondrial F0 complex, subunit G 5.24 0.0140 6.63 0.0034

NM_001697 ATP5O ATP synthase, mitochondrial F1 complex, O subunit 7.67 0.0011 7.62 0.0007

BC005876 ATP6V0B ATPase, lysosomal 21kDa, V0 subunit B 2.29 0.1201 3.78 0.0104

M62762 ATP6V0C ATPase, lysosomal 16kDa, V0 subunit C 3.27 0.0245 5.46 0.0019

AL566172 ATP6V0D1 ATPase, lysosomal 38kDa, V0 subunit D1 2.94 0.0405 3.53 0.0137

AI862255 ATP6V0E1 ATPase, lysosomal 9kDa, V0 subunit E1 12.38 0.0016 11.47 0.0012

AF113129 ATP6V1A ATPase, lysosomal 70kDa, V1 subunit A 2.37 0.0144 2.69 0.0043

AF100741 ATP6V1D ATPase, lysosomal 34kDa, V1 subunit D 2.58 0.0556 4.59 0.0029

NM_004231 ATP6V1F ATPase, lysosomal 14kDa, V1 subunit F 1.56 0.3701 2.36 0.0445

BF508371 TNFRSF1A Tumor necrosis factor receptor superfamily, member 1A  **-4.78** 0.0008  **-4.46** 0.0007

***B cell receptor signaling pathway***

NM_013314 BLNK B-cell linker 2.90 0.2600 6.10 0.0330

NM_004356 CD81 CD81 molecule 3.05 0.0168 3.41 0.0068

M31933 FCGR2B Fc fragment of IgG, low affinity IIb, receptor (CD32) 3.48 0.0343 9.12 0.0007

NM_003641 IFITM1 Interferon induced transmembrane protein 1 (9-27) 7.06 0.0592 129.7 0.0002

AU153366 IKBKB Inhibitor of kappa light polypeptide gene enhancer

in B-cells, kinase beta 3.48 0.0049 4.22 0.0012

AF074382 IKBKG Inhibitor of kappa light polypeptide gene enhancer

in B-cells, kinase gamma  **-2.09** 0.0011  **-2.02** 0.0010

W80678 KRAS v-Ki-ras2 Kirsten rat sarcoma viral oncogene homolog 4.40 0.0105 2.77 0.0520

W61007 NFAT5 Nuclear factor of activated T-cells 5, tonicity-responsive 4.40 0.0105 4.34 0.0015

AI078167 NFKBIA Nuclear factor of kappa light polypeptide gene enhancer

in B-cells inhibitor, alpha 2.32 0.0934 2.78 0.0313

BC004247 RAC1 Ras-related C3 botulinum toxin substrate 1

(rho family, small GTP binding protein Rac1) 3.11 0.0234 4.56 0.0029

AI753792 RRAS2 Related RAS viral (r-ras) oncogene homolog 2 2.05 0.0108 2.21 0.0040

AV682436 PIK3C2A Phosphoinositide-3-kinase, class 2, alpha polypeptide 2.60 0.0161 2.47 0.0150

AI679268 PIK3R1 Phosphoinositide-3-kinase, regulatory subunit 1 (p85 alpha) 3.83 0.0249 2.36 0.1320

BF740111 PIK3R4 Phosphoinositide-3-kinase, regulatory subunit 4, p150 2.71 0.0246 1.69 0.2200

***Biosynthesis of steroids***

BC003573 FDFT1 Farnesyl-diphosphate farnesyltransferase 1 5.69 0.0195 6.02 0.0118

NM_002004 FDPS Farnesyl diphosphate synthase

(farnesyl pyrophosphate synthetase, geranyltranstransferase) 3.63 0.0218 5.02 0.0041

AF098865 SQLE Squalene epoxidase 1.56 0.5090 3.29 0.0226

NM_024006 VKORC1 Vitamin K epoxide reductase complex, subunit 1 3.50 0.0319 2.68 0.0730

***C21-Steroid hormone metabolism***

AB045829 AKR1C4 Aldo-keto reductase family 1, member C4

(3-alpha hydroxysteroid dehydrogenase, type I) 10.62 0.0015 7.83 0.0025

NM_005525 HSD11B1 Hydroxysteroid (11-beta) dehydrogenase 1 2.78 0.0325 2.15 0.0874

***Calcium signaling pathway***

N51516 ADRA1A Adrenergic, alpha-1A-, receptor 6.27 0.0253 6.36 0.0176

NM_004835 AGTR1 Angiotensin II receptor, type 1 2.41 0.0005 1.61 0.0168

R52647 ATP2B2 ATPase, Ca++ transporting, plasma membrane 2 4.95 0.0042 1.95 0.1810

NM_021096 CACNA1 Calcium channel, voltage-dependent, alpha 1I subunit **-2.01** 0.0163 **-2.00** 0.0123

M27319 CALM1 Calmodulin 1 (phosphorylase kinase, delta) 9.18 0.0007 7.31 0.0010

NM_001743 CALM2 Calmodulin 2 (phosphorylase kinase, delta) 4.28 0.0235 4.99 0.0099

AA807959 CALM3 Calmodulin 3 (phosphorylase kinase, delta) 2.92 0.0030 3.70 0.0005

BF797381 CAMK2D Calcium/calmodulin-dependent protein kinase (CaM kinase) II delta 2.39 0.0875 3.68 0.0096

NM_004380 CREBBP CREB binding protein (Rubinstein-Taybi syndrome) 3.53 0.0017 4.69 0.0003

M74921 EDNRB Endothelin receptor type B 3.16 0.0645 8.39 0.0015

AI459462 EP300 E1A binding protein p300 2.54 0.0147 2.34 0.0124

AV681807 ERBB3 v-erb-b2 erythroblastic leukemia viral oncogene homolog 3 (avian) 2.75 0.0258 2.42 0.0382

BF222895 GNAQ Guanine nucleotide binding protein (G protein), q polypeptide 2.34 0.0171 2.42 0.0094

AF064092 GNAS GNAS complex locus 2.54 0.0318 2.44 0.0299

AA834576 ITPR2 Inositol 1,4,5-triphosphate receptor, type 2 9.51 0.0005 7.31 0.0008

NM_005965 MYLK Myosin, light chain kinase /// myosin, light chain kinase 3.63 0.0257 2.63 0.0740

W61007 NFAT5 Nuclear factor of activated T-cells 5, tonicity-responsive 4.37 0.0023 4.34 0.0015

AI078167 NFKBIA Nuclear factor of kappa light polypeptide gene enhancer

in B-cells inhibitor, alpha 2.32 0.0934 2.78 0.0313

NM_006206 PDGFRA Platelet-derived growth factor receptor, alpha polypeptide 3.23 0.1280 4.72 0.0302

AI014573 PPID Peptidylprolyl isomerase D (cyclophilin D) 2.14 0.2190 3.48 0.0278

AA130247 PRKACB Protein kinase, cAMP-dependent, catalytic, beta 2.53 0.0411 3.01 0.0124

NM_002863 PYGL Phosphorylase, glycogen; liver (Hers disease, glycogen storage disease type VI) 4.95 0.0120 3.13 0.0485

AA916851 SLC25A6 Solute carrier family 25 (adenine nucleotide translocator), member 6 3.45 0.0101 2.94 0.0158

NM_001222 SRP72 Signal recognition particle 72kDa 5.31 0.0014 5.38 0.0008

AL515918 VDAC1 Voltage-dependent anion channel 1 6.19 0.0208 7.94 0.0071

U90943 VDAC3 Voltage-dependent anion channel 3 (inositol 1,4,5-triphosphate receptor, type 2) 10.62 0.0018 10.62 0.0012

***Cell adhesion molecules (CAMs) and MHC receptors***

U82164 CD99 CD99 molecule 2.75 0.0297 2.23 0.0657

AL360136 CD276 CD276 molecule **-2.94** 0.0006 **-3.24** 0.0002

AF172398 F11R F11 receptor 2.65 0.0762 3.09 0.0303

AK025457 GLG1 Golgi apparatus protein 1 /// sema domain, transmembrane domain (TM), and

cytoplasmic domain, (semaphorin) 6C /// CDC42 small effector 1 3.65 0.0013 4.25 0.0004

AA573862 HLA-A Major histocompatibility complex, class I, A 3.09 0.1430 12.55 0.0016

L07950 HLA-B Major histocompatibility complex, class I, B 14.42 0.0066 76.63 0.0002

U62824 HLA-C Major histocompatibility complex, class I, C 2.08 0.3240 7.62 0.0035

M27487 HLA-DPA1 Major histocompatibility complex, class II, DP alpha 1 2.58 0.1710 10.26 0.0013

BG397856 HLA-DQA1-2 Major histocompatibility complex, class II, DQ alpha 1 and alpha 2 1.30 0.8190 3.50 0.0192

M60334 HLA-DRA Major histocompatibility complex, class II, DR alpha 6.02 0.0190 29.65 0.0002

U65585 HLA-DRB1 Major histocompatibility complex, class II, DR beta 1 3.58 0.1040 19.42 0.0007

AJ297586 HLA-DRB1-3 Major histocompatibility complex, class II, DR beta 1 and beta 3 1.53 0.6840 4.89 0.0168

X56841 HLA-E Major histocompatibility complex, class I, E 10.62 0.0002 23.91 0.0001

AW514210 HLA-F Major histocompatibility complex, class I, F 4.02 0.0862 18.37 0.0010

M90685 HLA-G HLA-G histocompatibility antigen, class I, G 3.68 0.0336 13.45 0.0003

NM_133376 ITGB1 Integrin, beta 1 (fibronectin receptor, beta polypeptide,

antigen CD29 includes MDF2, MSK12) 5.73 0.0098 6.19 0.0051

AI809341 PTPRC Protein tyrosine phosphatase, receptor type, C 1.55 0.6290 3.97 0.0264

AI520949 PVRL2 Poliovirus receptor-related 2 (herpesvirus entry mediator B) 2.12 0.0469 2.54 0.0117

AA129716 PVRL3 Poliovirus receptor-related 3 4.40 0.0332 4.00 0.0351

NM_002997 SDC1 Syndecan 1 3.18 0.0156 2.58 0.0319

AI380298 SDC2 Syndecan 2 (heparan sulfate proteoglycan 1,

cell surface-associated, fibroglycan) 6.14 0.0025 4.95 0.0038

***Cell cycle***

AI149508 ACVR2A Activin A receptor, type IIA 4.46 0.0014 3.24 0.0043

AU160695 BUB3 BUB3 budding uninhibited by benzimidazoles 3 homolog (yeast) 3.50 0.0004 3.36 0.0004

NM_000075 CDK4 Cyclin-dependent kinase 4 2.26 0.0166 2.94 0.0020

U17074 CDKN2C Cyclin-dependent kinase inhibitor 2C (p18, inhibits CDK4) **-3.16** 0.0002 **-3.18** 0.0001

AV727101 EP300 E1A binding protein p300 2.54 0.0147 2.47 0.0120

NM_001527 HDAC2 Histone deacetylase 2 2.69 0.0097 2.56 0.0090

NM_016596 HDAC7A Histone deacetylase 7A **-7.62** 0.0003 **-6.96** 0.0002

BG105365 MCAM Melanoma cell adhesion molecule 2.92 0.0083 2.08 0.0446

AF047598 ORC4L Origin recognition complex, subunit 4-like (yeast) 4.59 0.0041 4.31 0.0036

NM_002592 PCNA Proliferating cell nuclear antigen 3.07 0.0179 5.57 0.0008

NM_000321 RB1 Retinoblastoma 1 (including osteosarcoma) 2.41 0.0193 2.11 0.0321

***Citrate cycle (TCA cycle)***

NM_001096 ACLY ATP citrate lyase 2.14 0.0801 3.58 0.0040

J03620 DLD Dihydrolipoamide dehydrogenase 7.01 0.0149 7.01 0.0105

AI363836 FH Fumarate hydratase 5.54 0.0045 3.05 0.0356

AI826060 IDH3A Isocitrate dehydrogenase 3 (NAD+) alpha 1.51 0.4100 2.29 0.0440

AF023266 IDH3B Isocitrate dehydrogenase 3 (NAD+) beta 2.41 0.0219 2.18 0.0290

NM_004135 IDH3G Isocitrate dehydrogenase 3 (NAD+) gamma 2.14 0.0262 2.29 0.0122

NM_005917 MDH1 Malate dehydrogenase 1, NAD (soluble) 9.51 0.0057 9.25 0.0040

BC001917 MDH2 Malate dehydrogenase 2, NAD (mitochondrial) 2.77 0.0739 3.48 0.0221

NM_004168 SDHA Succinate dehydrogenase complex, A, flavoprotein 4.16 0.0108 4.02 0.0088

NM_003000 SDHB Succinate dehydrogenase complex, B, iron sulfur 5.97 0.0120 6.68 0.0056

AF080579 SDHC Succinate dehydrogenase complex, C, membrane protein 14.12 0.0003 11.63 0.0004

AL050226 SUCLG2 Succinate-CoA ligase, GDP-forming, beta subunit 4.22 0.0108 2.82 0.0442

***Complement and coagulation cascades***

NM_000014 A2M Alpha-2-macroglobulin 3.31 0.0219 2.71 0.0417

NM_000491 C1QB Complement component 1, q subcomponent, B chain 3.65 0.1140 12.2 0.0029

AI184968 C1QC Complement component 1, q subcomponent, C chain 1.47 0.5402 3.27 0.0140

AL573058 C1R Complement component 1, r subcomponent 2.20 0.0287 2.73 0.0054

BC007010 C1S Complement component 1, s subcomponent 53.44 0.0006 50.21 0.0004

NM_000592 C4A/C4B Complement component 4A (Rodgers blood group) ///

complement component 4B (Childo blood group) 5.20 0.0002 6.32 0.0001

NM_000716 C4BPB Complement component 4 binding protein, beta 3.22 0.0401 5.65 0.0032

NM_001735 C5 Complement component 5 8.75 0.0015 7.83 0.0013

J05064 C6 Complement component 6 3.24 0.0332 2.63 0.0624

NM_000587 C7 Complement component 7 2.34 0.1850 4.28 0.0152

M17263 C8G Complement component 8, gamma polypeptide 4.72 0.0051 4.56 0.0038

AL570661 CD46 CD46 molecule, complement regulatory protein 4.72 0.0015 3.24 0.0060

X04697 CFH Complement factor H 2.90 0.0124 2.86 0.0091

NM_000506 F2 Coagulation factor II (thrombin) 2.11 0.0329 1.65 0.1360

AA910306 F5 Coagulation factor V (proaccelerin, labile factor) 3.16 0.0218 2.47 0.0071

NM_000504 F10 Coagulation factor X 2.42 0.0070 1.85 0.0513

NM_000128 F11 Coagulation factor XI (plasma thromboplastin antecedent) **-2.28** 0.0150 **-2.75** 0.0029

NM_021871 FGA Fibrinogen alpha chain 3.86 0.0035 3.48 0.0039

BG545288 FGB Fibrinogen beta chain 49.86 0.0001 45.56 0.0001

NM_000509 FGG Fibrinogen gamma chain 2.67 0.0355 2.56 0.0325

M74220 LPA/PLG Lipoprotein, Lp(a) /// plasminogen 4.69 0.0256 3.81 0.0396

NM_000892 KLKB1 Kallikrein B, plasma (Fletcher factor) 1  **-1.70** 0.1730 **-2.32** 0.0207

AI274095 MASP1 Mannan-binding lectin serine peptidase 1

(C4/C2 activating component of Ra-reactive factor) 3.07 0.0004 2.78 0.0005

AB008047 MASP2 Mannan-binding lectin serine peptidase 2 4.75 0.0144 3.16 0.0489

NM_000242 MBL2 Mannose-binding lectin (protein C) 2, soluble (opsonic defect) 3.18 0.0162 1.37 0.5910

BE880828 MCFD2 Multiple coagulation factor deficiency 2 11.71 0.0004 14.72 0.0001

M74220 PLG Plasminogen 3.86 0.0059 3.60 0.0054

NM_000295 SERPINA1 Serpin peptidase inhibitor, clade A (alpha-1 antiproteinase, antitrypsin), member 1 8.20 0.0003 7.21 0.0004

BC022309 SERPINC1 Serpin peptidase inhibitor, clade C (antithrombin), member 1 30.65 0.0005 19.29 0.0009

NM_000062 SERPING1 Serpin peptidase inhibitor, clade G (C1 inhibitor), member 1 2.77 0.0336 3.07 0.0296

BF511231 TFPI Tissue factor pathway inhibitor (lipoprotein-associated coagulation inhibitor) 2.05 0.0312 1.45 0.2590

***Cytokine-cytokine receptor interaction***

NM_001105 ACVR1 Activin A receptor, type I 10.85 0.0008 7.62 0.0016

AI457436 BMPR2 Bone morphogenetic protein receptor, type II (serine/threonine kinase) 4.95 0.0066 2.82 0.0473

M57731 CXCL2 Chemokine (C-X-C motif) ligand 2 1.55 0.5540 4.50 0.0090

NM_001565 CXCL10 Chemokine (C-X-C motif) ligand 10 1.72 0.7500 17.14 0.0047

U19495 CXCL12 Chemokine (C-X-C motif) ligand 12 (stromal cell-derived factor 1) 5.61 0.0035 6.02 0.0017

U62858 IL13RA1 Interleukin 13 receptor, alpha 1 /// interleukin 13 receptor, alpha 1 3.65 0.0137 4.59 0.0034

AK026803 IL1R1 Interleukin 1 receptor, type I 6.10 0.0046 8.51 0.0010

U64094 IL1R2 Interleukin 1 receptor, type II 4.16 0.0293 6.77 0.0040

AV700030 IL6R Interleukin 6 receptor 3.91 0.0312 3.91 0.0231

AB015706 IL6ST Interleukin 6 signal transducer (gp130, oncostatin M receptor) 6.27 0.0580 10.26 0.0136

NM_000880 IL7 Interleukin 7 4.69 0.0366 12.99 0.0013

NM_005538 INHBC Inhibin, beta C 6.58 0.0035 5.38 0.0048

AI680541 LIFR Leukemia inhibitory factor receptor alpha 7.21 0.0028 5.65 0.0043

NM_006206 PDGFRA Platelet-derived growth factor receptor, alpha polypeptide 3.22 0.1280 4.72 0.0302

NM_016205 PDGFC Platelet derived growth factor C 2.17 0.0460 1.84 0.1030

AA604375 TGFBR1 Transforming growth factor, beta receptor I 4.28 0.0083 3.09 0.0238

D50683 TGFBR2 Transforming growth factor, beta receptor II (70/80kDa) 2.09 0.0726 2.37 0.0275

NM_003810 TNFSF10 Tumor necrosis factor (ligand) superfamily, member 10 8.00 0.0062 9.51 0.0024

AF134715 TNFSF13B Tumor necrosis factor (ligand) superfamily, member 13b 1.00 0.1000 3.75 0.0009

AF022375 VEGFA Vascular endothelial growth factor A 4.95 0.0054 3.43 0.0171

***Fatty acid metabolism***

AI057637 ACACB Acetyl-Coenzyme A carboxylase beta  **-3.58** 0.0103  **-4.38** 0.0027

BC000408 ACAT2 Acetyl-Coenzyme A acetyltransferase 2 (acetoacetyl Coenzyme A thiolase) 6.10 0.0065 4.78 0.0107

NM_003500 ACOX2 Acyl-Coenzyme A oxidase 2, branched chain 6.63 0.0019 3.11 0.0267

NM_001995 ACSL1 Acyl-CoA synthetase long-chain family member 1 9.91 0.0016 5.13 0.0091

D89053 ACSL3 Acyl-CoA synthetase long-chain family member 3 5.97 0.0060 6.77 0.0025

AW173691 ACSL5 acyl-CoA synthetase long-chain family member 5 2.11 0.0922 2.69 0.0199

AV651117 ADH4 Alcohol dehydrogenase 4 (class II), pi polypeptide 5.85 0.0116 2.62 0.1390

BC002430 ALDH3A2 Aldehyde dehydrogenase 3 family, member A2 3.03 0.0194 2.14 0.0831

BC002515 ALDH7A1 Aldehyde dehydrogenase 7 family, member A1 6.06 0.0028 2.17 0.1410

AF182275 CYP2A6 Cytochrome P450, family 2, subfamily A, polypeptide 6 17.87 0.0008 7.21 0.0009

NM_000764 CYP2A13 Cytochrome P450, family 2, subfamily A, polypeptide 13 6.45 0.0008 2.88 0.0186

X06399 CYP2B6 Cytochrome P450, family 2, subfamily B, polypeptide 6 3.16 0.0007 -1.02 0.9980

NM_030878 CYP2C8 Cytochrome P450, family 2, subfamily C, polypeptide 8 3.50 0.0020 2.34 0.0147

NM_000106 CYP2D6 Cytochrome P450, family 2, subfamily D, polypeptide 6 7.78 0.0065 2.65 0.1530

AF182276 CYP2E1 Cytochrome P450, family 2, subfamily E, polypeptide 1 4.05 0.0150 2.86 0.0459

NM_000777 CYP3A5 Cytochrome P450, family 3, subfamily A, polypeptide 5 7.56 0.0005 6.36 0.0006

AF315325 CYP3A7 Cytochrome P450, family 3, subfamily A, polypeptide 7 11.39 0.0006 5.27 0.0050

AF280111 CYP3A43 Cytochrome P450, family 3, subfamily A, polypeptide 43 11.63 0.0029 6.06 0.0131

NM_004092 ECHS1 Enoyl Coenzyme A hydratase, short chain, 1, mitochondrial 7.06 0.0113 4.78 0.0262

NM_000159 GCDH Glutaryl-Coenzyme A dehydrogenase 2.26 0.0312 1.66 0.1691

U04627 HADHA Hydroxyacyl-Coenzyme A dehydrogenase/ 3-ketoacyl-Coenzyme A thiolase/

enoyl-Coenzyme A hydratase (trifunctional protein), alpha subunit 3.43 0.0180 3.75 0.0086

NM_006117 PECI Peroxisomal D3,D2-enoyl-CoA isomerase 8.05 0.0025 4.56 0.0122

BF224073 TCP1 T-complex 1 3.43 0.0332 2.49 0.0977

***Fatty acid biosynthesis (path 2)***

BC000408 ACAT2 Acetyl-Coenzyme A acetyltransferase 2 (acetoacetyl Coenzyme A thiolase) 6.10 0.0065 4.78 0.0107

NM_004092 ECHS1 Enoyl Coenzyme A hydratase, short chain, 1, mitochondrial 7.06 0.0113 4.78 0.0262

NM_014762 DHCR24 24-dehydrocholesterol reductase 5.27 0.0006 3.65 0.0021

U04627 HADHA Hydroxyacyl-Coenzyme A dehydrogenase/ 3-ketoacyl-Coenzyme A thiolase/

enoyl-Coenzyme A hydratase (trifunctional protein), alpha subunit 3.43 0.0180 3.75 0.0086

BF224073 TCP1 T-complex 1 3.43 0.0332 2.49 0.0977

***Glycolysis / Gluconeogenesis***

AV651117 ADH4 Alcohol dehydrogenase 4 (class II), pi polypeptide 5.85 0.0116 2.62 0.1390

BC002430 ALDH3A2 Aldehyde dehydrogenase 3 family, member A2 3.03 0.0194 2.14 0.0831

BC002515 ALDH7A1 Aldehyde dehydrogenase 7 family, member A1 6.06 0.0028 2.17 0.1410

NM_000034 ALDOA Aldolase A, fructose-bisphosphate 2.02 0.0136 3.05 0.0005

AK026411 ALDOB Aldolase B, fructose-bisphosphate 14.84 0.0003 6.02 0.0028

NM_000035 ALDOB Aldolase B, fructose-bisphosphate 6.32 0.0033 3.01 0.0423

J03620 DLD Dihydrolipoamide dehydrogenase 7.01 0.0149 7.01 0.0105

D26054 FBP1 Fructose-1,6-bisphosphatase 1 4.08 0.0062 1.59 0.3590

BC020700 G6PC Glucose-6-phosphatase, catalytic subunit 12.17 0.0035 5.16 0.0265

AK026525 GAPDH Glyceraldehyde-3-phosphate dehydrogenase 6.58 0.0035 4.89 0.0066

AI769923 GALM Galactose mutarotase (aldose 1-epimerase)  **-1.75** 0.0469  **-2.05** 0.0095

M69051 GCK Glucokinase (hexokinase 4,

maturity onset diabetes of the young 2) 3.91 0.0001 **- 1.01** 0.9900

NM_000175 GPI Glucose phosphate isomerase 4.14 0.0020 4.31 0.0010

NM_000284 PDHA1 Pyruvate dehydrogenase (lipoamide) alpha 1 3.48 0.0769 4.53 0.0249

S81916 PGK1 Phosphoglycerate kinase 1 23.58 0.0009 23.42 0.0006

NM_002633 PGM1 Phosphoglucomutase 1 6.27 0.0039 5.02 0.0058

AV727934 PGM3 Phosphoglucomutase 3 3.45 0.0339 2.08 0.0200

BF116254 TPI1 Triosephosphate isomerase 1 2.18 0.0173 2.12 0.0146

***Glyoxylate and dicarboxylate metabolism***

NM_012203 GRHPR Glyoxylate reductase/hydroxypyruvate reductase 5.73 0.0054 2.80 0.0616

AF284751 HYI Hydroxypyruvate isomerase homolog (E. coli) 2.29 0.0129 1.55 0.1590

NM_005917 MDH1 Malate dehydrogenase 1, NAD (soluble) 9.51 0.0057 9.25 0.0039

BC001917 MDH2 Malate dehydrogenase 2, NAD (mitochondrial) 2.77 0.0739 3.48 0.0221

***Hematopoietic cell lineage***

NM_001769 CD9 CD9 molecule 9.38 0.0147 9.25 0.0107

NM_000072 CD36 CD36 molecule (thrombospondin receptor) 47.17 0.0001 16.11 0.0008

M60334 HLA-DRA Major histocompatibility complex, class II, DR alpha 6.02 0.0190 29.65 0.0002

AK026803 IL1R1 Interleukin 1 receptor, type I 6.10 0.0046 8.51 0.0010

U64094 IL1R2 Interleukin 1 receptor, type II 4.16 0.0293 6.77 0.0040

AV700030 IL6R Interleukin 6 receptor 3.91 0.0312 3.91 0.0231

NM_000880 IL7 Interleukin 7 4.69 0.0366 12.99 0.0013

***Hedgehog signaling pathway***

BF341845 CSNK1A1 Casein kinase 1, alpha 1 6.23 0.0138 7.21 0.0060

NM_001893 CSNK1D Casein kinase 1, delta 2.53 0.0427 3.27 0.0086

AA130247 PRKACB Protein kinase, cAMP-dependent, catalytic, beta 2.53 0.0411 3.01 0.0124

NM_005631 SMO Smoothened homolog (Drosophila)  **-2.29** 0.0437  **-2.29** 0.0342

***Insulin signaling pathway***

AI057637 ACACB Acetyl-Coenzyme A carboxylase beta  **-3.58** 0.0103  **-4.38** 0.0027

M27319 CALM1 Calmodulin 1 (phosphorylase kinase, delta) 9.18 0.0007 7.31 0.0010

NM_001743 CALM2 Calmodulin 2 (phosphorylase kinase, delta) 4.28 0.0235 4.99 0.0099

AA807959 CALM3 Calmodulin 3 (phosphorylase kinase, delta) 2.92 0.0030 3.70 0.0005

AW268640 EIF4E Eukaryotic translation initiation factor 4E 5.27 0.0045 6.06 0.0017

D26054 FBP1 Fructose-1,6-bisphosphatase 1 4.08 0.0062 1.59 0.3500

M69051 GCK Glucokinase (hexokinase 4,

maturity onset diabetes of the young 2) 3.91 0.0001  **-1.01** 0.9900

BC020700 G6PC Glucose-6-phosphatase, catalytic subunit 12.17 0.0035 5.16 0.0265

S70004 GYS2 Glycogen synthase 2 (liver) 9.64 0.0005 5.16 0.0265

AU153366 IKBKB Inhibitor of kappa light polypeptide gene enhancer in B-cells, kinase beta 3.48 0.0049 4.22 0.0012

NM_005544 IRS1 Insulin receptor substrate 1 2.07 0.0221 1.46 0.2180

W80678 KRAS v-Ki-ras2 Kirsten rat sarcoma

viral oncogene homolog 4.40 0.0105 2.76 0.0520

NM_017572 MKNK2 MAP kinase interacting serine/threonine kinase 2 2.62 0.0109 2.84 0.0045

AI679268 PIK3R1 Phosphoinositide-3-kinase, regulatory subunit 1 (p85 alpha) 3.83 0.0249 2.36 0.1320

BF740111 PIK3R4 phosphoinositide-3-kinase, regulatory subunit 4, p150 2.71 0.0246 1.69 0.2230

NM_002708 PPP1CA Protein phosphatase 1, catalytic subunit, alpha 8.69 0.0001 7.94 0.0001

N26005 PPP1R3C Protein phosphatase 1, regulatory (inhibitor) subunit 3C 25.28 0.0014 9.98 0.0083

AL552001 PRKAB2 Protein kinase, AMP-activated, beta 2 2.65 0.0108 2.00 0.0467

AA130247 PRKACB Protein kinase, cAMP-dependent, catalytic, beta 2.53 0.0411 3.01 0.0124

NM_002734 PRKAR1A Protein kinase, cAMP-dependent, regulatory, type I, alpha 5.89 0.0128 4.40 0.0240

AK026351 PRKAR2A Protein kinase, cAMP-dependent, regulatory, type II, alpha 3.11 0.0127 3.13 0.0485

NM_002863 PYGL Phosphorylase, glycogen; liver

(Hers disease, glycogen storage disease type VI) 4.95 0.0120 3.13 0.0485

NM_002880 RAF1 v-raf-1 murine leukemia viral oncogene homolog 1 3.41 0.0064 3.65 0.0030

AF493921 RHEB Ras homolog enriched in brain 5.42 0.0127 7.11 0.0036

BE741754 RPS6 Ribosomal protein S6 3.29 0.0428 3.38 0.0023

M60725 RPS6KB1 Ribosomal protein S6 kinase, 70kDa, polypeptide 1 2.63 0.0141 3.38 0.0023

AI753792 RRAS2 Related RAS viral (r-ras) oncogene homolog 2 2.05 0.0108 2.21 0.0040

AI091079 SHC1 SHC (Src homology 2 domain containing) transforming protein 1 2.78 0.0817 3.34 0.0313

AB004903 SOCS2 Suppressor of cytokine signaling 2 7.41 0.0086 2.71 0.1530

AU157543 SOCS6 CDNA FLJ14294 fis, clone PLACE1008181 4.43 0.0002 3.63 0.0004

AF013168 TSC1 Tuberous sclerosis 1 2.07 0.1140 2.86 0.0173

***Jak-STAT signaling pathway***

NM_004380 CREBBP CREB binding protein (Rubinstein-Taybi syndrome) 3.53 0.0017 4.69 0.0003

AV727101 EP300 E1A binding protein p300 2.54 0.0147 2.47 0.0120

L29511 GRB2 Growth factor receptor-bound protein 2 1.13 0.8380 2.09 0.0054

U62858 IL13RA1 Interleukin 13 receptor, alpha 1 3.65 0.0137 4.59 0.0034

AV700030 IL6R Interleukin 6 receptor 3.91 0.0312 3.91 0.0231

AB015706 IL6ST Interleukin 6 signal transducer (gp130, oncostatin M receptor) 6.27 0.0580 10.26 0.0136

NM_000880 IL7 Interleukin 7 4.69 0.0366 12.99 0.0013

U50748 LEPR Leptin receptor  **-1.36** 0.8270 **-3.68** 0.0426

AI680541 LIFR Leukemia inhibitory factor receptor alpha 7.21 0.0028 5.65 0.0043

AV682436 PIK3C2A Phosphoinositide-3-kinase, class 2, alpha polypeptide 2.60 0.0161 2.47 0.0150

U79291 PTPN11 Protein tyrosine phosphatase, non-receptor type 11 3.38 0.0235 3.89 0.0091

AU157543 SOCS6 CDNA FLJ14294 fis, clone PLACE1008181 4.43 0.0002 3.63 0.0004

BE967019 SPRED1 Sprouty-related, EVH1 domain containing 1 2.86 0.0478 3.41 0.0170

BC002704 STAT1 Signal transducer and activator of transcription 1, 91kDa 6.82 0.2001 49.52 0.0077

H98105 STAT2 Signal transducer and activator of transcription 2, 113kDa 3.22 0.0030 4.89 0.0002

AA634272 STAT3 Signal transducer and activator of transcription 3 3.55 0.2001 8.57 0.0185

***MAPK signaling pathway***

AL832061 ARRB2 Arrestin, beta 2  **-2.15** 0.0374  **-1.97** 0.0503

BE786164 ATF2 Activating transcription factor 2 5.31 0.0004 4.85 0.0004

M87507 CASP1 Caspase 1, apoptosis-related cysteine peptidase 2.04 0.0269 2.84 0.0019

NM_000591 CD14 CD14 molecule 3.22 0.0496 1.57 0.5290

N92917 CDC42 Cell division cycle 42 (GTP binding protein, 25kDa) 2.78 0.1931 4.19 0.0451

BC003143 DUSP6 Dual specificity phosphatase 6 5.69 0.0077 3.29 0.0412

NM_005345 HSPA1A-1B Heat shock 70kDa protein 1A-1B 1.91 0.5301 7.36 0.0126

AF216292 HSPA5 Heat shock 70kDa protein 5 (glucose-regulated protein, 78kDa) 19.15 0.0029 15.34 0.0032

AB034951 HSPA8 Heat shock 70kDa protein 8 82.13 0.0001 114.5 0.0001

NM_004134 HSPA9 Heat shock 70kDa protein 9 (mortalin) 4.28 0.0198 5.61 0.0052

NM_001540 HSPB1 Heat shock 27kDa protein 1 28.64 0.0008 36.75 0.0003

AU153366 IKBKB Inhibitor of kappa light polypeptide gene enhancer in B-cells, kinase beta 3.48 0.0049 4.22 0.0012

AF074382 IKBKG Inhibitor of kappa light polypeptide gene enhancer

in B-cells, kinase gamma  **-2.09** 0.0011 **-2.02** 0.0010

AK026803 IL1R1 Interleukin 1 receptor, type I 6.10 0.0046 8.51 0.0010

U64094 IL1R2 Interleukin 1 receptor, type II 4.16 0.0293 6.77 0.0040

NM_005354 JUND Jun D proto-oncogene 3.19 0.0936 4.25 0.0278

W80678 KRAS v-Ki-ras2 Kirsten rat sarcoma viral oncogene homolog 4.40 0.0105 2.77 0.0520

BC005365 MAP2K7 Mitogen-activated protein kinase kinase 7  **-2.26** 0.0079  **-2.11** 0.0092

AA541479 MAP3K1 Mitogen-activated protein kinase kinase kinase 1 2.00 0.1550E 2.71 0.0266

NM_004721 MAP3K13 Mitogen-activated protein kinase kinase kinase 13 2.49 0.0281 2.63 0.0146

NM_003618 MAP4K3 Mitogen-activated protein kinase kinase kinase kinase 3 2.67 0.0019 2.88 0.0007

NM_017572 MKNK2 MAP kinase interacting serine/threonine kinase 2 2.62 0.0109 2.84 0.0045

AW054826 NF1 Neurofibromin 1 2.02 0.0283

NM_006206 PDGFRA Platelet-derived growth factor receptor, alpha polypeptide 3.23 0.1280 4.72 0.0302

NM_000300 PLA2G2A Phospholipase A2, group IIA (platelets, synovial fluid) 2.18 0.7700 26.17 0.0218

AJ271832 PPM1B Protein phosphatase 1B (formerly 2C), magnesium-dependent, beta isoform 2.40 0.1940 3.73 0.0329

AA130247 PRKACB Protein kinase, cAMP-dependent, catalytic, beta 2.53 0.0411 3.01 0.0124

BC004247 RAC1 Ras-related C3 botulinum toxin substrate 1 (rho family) 3.11 0.0234 4.56 0.0029

NM_002880 RAF1 v-raf-1 murine leukemia viral oncogene homolog 1 3.41 0.0064 3.65 0.0030

NM_002890 RASA1 RAS p21 protein activator (GTPase activating protein) 1 2.31 0.0268 2.36 0.0175

AA906056 RPS6KA3 Ribosomal protein S6 kinase, 90kDa, polypeptide 3 3.14 0.0509 5.38 0.0045

AI753792 RRAS2 Related RAS viral (r-ras) oncogene homolog 2 2.05 0.0108 2.21 0.0040

AA604375 TGFBR1 Transforming growth factor, beta receptor I 4.28 0.0083 3.09 0.0238

D50683 TGFBR2 Transforming growth factor, beta receptor II (70/80kDa) 2.10 0.0726 2.37 0.0275

BF508371 TNFRSF1A Tumor necrosis factor receptor superfamily, member 1A  **-4.78** 0.0008  **-4.46** 0.0007

***Nitrogen metabolism***

W80357 CPS1 Carbamoyl-phosphate synthetase 1, mitochondrial 41.93 0.0008 31.55 0.0008

NM_001902 CTH Cystathionase (cystathionine gamma-lyase) 6.45 0.0316 5.89 0.0304

AF110329 GLS2 Glutaminase 2 (liver, mitochondrial) 30.06 0.0040 11.31 0.0201

AI339331 GLUD1 Glutamate dehydrogenase 1 2.35 0.0604 2.60 0.0264

AC006144 GLUD2 Glutamate dehydrogenase 2 11.08 0.0016 10.26 0.0012

NM_002065 GLUL Glutamate-ammonia ligase (glutamine synthetase) 6.63 0.0159 4.14 0.0483

***Notch signaling pathway***

NM_004380 CREBBP CREB binding protein (Rubinstein-Taybi syndrome) 3.53 0.0017 4.69 0.0003

AA053830 CTBP1 C-terminal binding protein 1 8.33 0.0054 7.06 0.0059

AA577672 DTX3L Deltex 3-like (Drosophila) 3.36 0.0686 6.96 0.0041

AV727101 EP300 E1A binding protein p300 2.54 0.0147 2.47 0.0120

NM_001527 HDAC2 Histone deacetylase 2 2.69 0.0097 2.56 0.0090

AI457817 JAG1 Jagged 1 (Alagille syndrome) 4.62 0.0154 6.40 0.0032

NM_018468 PSENEN Presenilin enhancer 2 homolog (C. elegans) 2.31 0.0210 3.09 0.0025

***Pentose phosphate pathway***

NM_000034 ALDOA Aldolase A, fructose-bisphosphate 2.02 0.0136 3.05 0.0005

AK026411 ALDOB Aldolase B, fructose-bisphosphate 14.82 0.0003 6.02 0.0028

D26054 FBP1 Fructose-1,6-bisphosphatase 1 4.08 0.0062 1.59 0.3590

NM_000175 GPI Glucose phosphate isomerase 4.14 0.0020 4.31 0.0010

NM_012088 PGLS Phosphoglucomutase 1 6.27 0.0039 5.02 0.0058

NM_006755 TALDO1 Transaldolase 1 3.60 0.0129 3.18 0.0158

L12711 TKT Transketolase (Wernicke-Korsakoff syndrome) 8.28 0.0022 7.51 0.0018

***Prostaglandin and leukotriene metabolism***

AB018580 AKR1C3 Aldo-keto reductase family 1, member C3 2.23 0.0463 2.11 0.0497

BC002511 CBR1 Carbonyl reductase 1 4.89 0.0221 2.11 0.2770

D26480 CYP4F2 Cytochrome P450, family 4, subfamily F, polypeptide 2 2.56 0.0002 2.23 0.0004

D12620 CYP4F3 Cytochrome P450, family 4, subfamily F, polypeptide 3 11.55 0.0005 9.64 0.0005

NM_021004 DHRS4 Dehydrogenase/reductase (SDR family) member 4 5.13 0.0043 2.16 0.1300

J02959 LTA4H Leukotriene A4 hydrolase 2.08 0.0462 1.75 0.1150

BE566894 LTB4DH Leukotriene B4 12-hydroxydehydrogenase 23.92 0.0021 8.46 0.0165

NM_000300 PLA2G2A Phospholipase A2, group IIA (platelets, synovial fluid) 2.19 0.7701 26.17 0.0218

***Pyruvate metabolism***

AI057637 ACACB Acetyl-Coenzyme A carboxylase beta  **-3.58** 0.0103  **-4.38** 0.0027

BC000408 ACAT2 Acetyl-Coenzyme A acetyltransferase 2 (acetoacetyl Coenzyme A thiolase) 6.10 0.0065 4.78 0.0107

BC002430 ALDH3A2 Aldehyde dehydrogenase 3 family, member A2 3.03 0.0194 2.14 0.0831

BC002515 ALDH7A1 Aldehyde dehydrogenase 7 family, member A1 6.06 0.0028 2.17 0.1401

J03620 DLD Dihydrolipoamide dehydrogenase 7.01 0.0149 7.01 0.0105

AK026752 GRHPR Glyoxylate reductase/hydroxypyruvate reductase 4.00 0.0087 1.99 0.1158

NM_005326 HAGH Hydroxyacylglutathione hydrolase 2.69 0.0158 2.00 0.0652

BC004353 HAGHL Hydroxyacylglutathione hydrolase-like  **-2.35** 0.0001  **-2.45** 0.0005

NM_005917 MDH1 Malate dehydrogenase 1, NAD (soluble) 9.51 0.0056 9.25 0.0040

BF224073 TCP1 t-complex 1 3.43 0.0332 2.50 0.0977

***Proteasome***

NM_002786 PSMA1 Proteasome (prosome, macropain) subunit, alpha type, 1 3.48 0.0495 3.70 0.0297

NM_002787 PSMA2 Proteasome (prosome, macropain) subunit, alpha type, 2 18.12 0.0039 22.16 0.0016

NM_002788 PSMA3 Proteasome (prosome, macropain) subunit, alpha type, 3 8.22 0.0025 10.19 0.0008

NM_002789 PSMA4 Proteasome (prosome, macropain) subunit, alpha type, 4 1.75 0.2681 2.73 0.0267

NM_002790 PSMA5 Proteasome (prosome, macropain) subunit, alpha type, 5 4.19 0.0054 4.43 0.0028

BC002979 PSMA6 Proteasome (prosome, macropain) subunit, alpha type, 6 9.51 0.0021 13.64 0.0005

NM_002792 PSMA7 Proteasome (prosome, macropain) subunit, alpha type, 7 5.09 0.0204 6.06 0.0082

NM_002793 PSMB1 Proteasome (prosome, macropain) subunit, beta type, 1 7.21 0.0101 7.26 0.0066

NM_002794 PSMB2 Proteasome (prosome, macropain) subunit, beta type, 2 4.59 0.0208 7.62 0.0026

NM_002795 PSMB3 Proteasome (prosome, macropain) subunit, beta type, 3 7.01 0.0005 9.51 0.0001

NM_002796 PSMB4 Proteasome (prosome, macropain) subunit, beta type, 4 7.26 0.0075 6.91 0.0058

NM_002799 PSMB7 Proteasome (prosome, macropain) subunit, beta type, 7 3.89 0.0404 4.46 0.0183

NM_002802 PSMC1 Proteasome (prosome, macropain) 26S subunit, ATPase, 1 3.48 0.0018 3.22 0.0017

NM_002803 PSMC2 Proteasome (prosome, macropain) 26S subunit, ATPase, 2 5.97 0.0238 6.06 0.0165

AL545523 PSMC3 Proteasome (prosome, macropain) 26S subunit, ATPase, 3 4.43 0.0213 3.36 0.0437

NM_002806 PSMC6 Proteasome (prosome, macropain) 26S subunit, ATPase, 6 3.41 0.0317 3.86 0.0141

AI860431 PSMD1 Proteasome (prosome, macropain) 26S subunit, non-ATPase, 1 2.62 0.0120 2.69 0.0070

NM_002809 PSMD3 Proteasome (prosome, macropain) 26S subunit, non-ATPase, 3 3.20 0.0008 3.45 0.0003

AB033605 PSMD4 Proteasome (prosome, macropain) 26S subunit, non-ATPase, 4 9.51 0.0004 11.95 0.0001

NM_014814 PSMD6 Proteasome (prosome, macropain) 26S subunit, non-ATPase, 6 6.86 0.0052 5.09 0.0098

AF001212 PSMD11 Proteasome (prosome, macropain) 26S subunit, non-ATPase, 11 5.46 0.0020 7.62 0.0004

AI446530 PSMD12 Proteasome (prosome, macropain) 26S subunit, non-ATPase, 12 4.08 0.0211 4.56 0.0098

***Synthesis and degradation of ketone bodies***

BC000408 ACAT2 Acetyl-Coenzyme A acetyltransferase 2 (acetoacetyl Coenzyme A thiolase) 6.10 0.0065 4.78 0.0107

BG035985 HMGCS1 3-hydroxy-3-methylglutaryl-Coenzyme A synthase 1 (soluble) 3.01 0.1981 4.69 0.0467

NM_005518 HMGCS2 3-hydroxy-3-methylglutaryl-Coenzyme A synthase 2 (mitochondrial) 5.16 0.0135 2.90 0.0782

BF224073 TCP1 t-complex 1 3.43 0.0332 2.50 0.0977

***T cell receptor signaling pathway***

N92917 CDC42 Cell division cycle 42 (GTP binding protein, 25kDa) 2.78 0.1930 4.19 0.0451

NM_000075 CDK4 Cyclin-dependent kinase 4 2.26 0.0166 2.94 0.0020

L29511 GRB2 Growth factor receptor-bound protein 2 1.12 0.8380 2.09 0.0054

AU153366 IKBKB Inhibitor of kappa light polypeptide gene enhancer in B-cells, kinase beta 3.48 0.0049 4.22 0.0012

W80678 KRAS v-Ki-ras2 Kirsten rat sarcoma viral oncogene homolog 4.40 0.0105 2.77 0.0520

NM_006153 NCK1 NCK adaptor protein 1 2.49 0.0103

W61007 NFAT5 Nuclear factor of activated T-cells 5, tonicity-responsive 4.40 0.0105 4.34 0.0015

AI078167 NFKBIA Nuclear factor of kappa light polypeptide gene enhancer

in B-cells inhibitor, alpha 2.32 0.0934 2.78 0.0313

AV682436 PIK3C2A Phosphoinositide-3-kinase, class 2, alpha polypeptide 2.60 0.0161 2.47 0.0150

AI679268 PIK3R1 Phosphoinositide-3-kinase, regulatory subunit 1 (p85 alpha) 3.83 0.0249 2.36 0.1320

BF740111 PIK3R4 Phosphoinositide-3-kinase, regulatory subunit 4, p150 2.71 0.0246 1.69 0.2200

AI809341 PTPRC Protein tyrosine phosphatase, receptor type, C 1.55 0.6290 3.97 0.0264

BC001360 RHOA Ras homolog gene family, member A 5.20 0.0142 4.78 0.0133

AI753792 RRAS2 Related RAS viral (r-ras) oncogene homolog 2 2.05 0.0108 2.21 0.0040

***TGF-beta signaling pathway***

NM_001105 ACVR1 Activin A receptor, type I 10.85 0.0008 7.62 0.0016

AI457436 BMPR2 Bone morphogenetic protein receptor, type II (serine/threonine kinase) 4.95 0.0066 2.82 0.0473

NM_004380 CREBBP CREB binding protein (Rubinstein-Taybi syndrome) 3.53 0.0017 4.69 0.0003

NM_003592 CUL1 Cullin 1 4.59 0.0120 4.08 0.0133

AF138302 DCN Decorin 27.85 0.0001 40.50 0.0000

AF138303 DCN Decorin 13.08 0.0002 19.15 0.0000

AV727101 EP300 E1A binding protein p300 2.54 0.0147 2.47 0.0120

NM_002166 ID2 Inhibitor of DNA binding 2, dominant negative helix-loop-helix protein 3.09 0.0549 4.22 0.0123

NM_005538 INHBC Inhibin, beta C 6.58 0.0035 5.38 0.0048

BC000400 PPP2CA Protein phosphatase 2 (formerly 2A), catalytic subunit, alpha isoform 8.93 0.0020 9.31 0.0011

NM_004156 PPP2CB Protein phosphatase 2 (formerly 2A), catalytic subunit, beta isoform 3.58 0.0865 4.31 0.0375

NM_014248 RBX1 Ring-box 1 2.51 0.0525 3.65 0.0065

BC001360 RHOA Ras homolog gene family, member A 5.20 0.0142 4.78 0.0133

M60725 RPS6KB1 Ribosomal protein S6 kinase, 70kDa, polypeptide 1 2.63 0.0141 3.38 0.0023

NM_006930 SKP1A S-phase kinase-associated protein 1A (p19A) 12.46 0.0030 15.03 0.0012

AU146891 SMAD1 SMAD family member 1 2.49 0.0392 2.82 0.0154

U65019 SMAD2 SMAD family member 2 2.20 0.1550 2.88 0.0387

AI478523 SMAD5 SMAD family member 5 2.82 0.0005 3.01 0.0002

AA604375 TGFBR1 Transforming growth factor, beta receptor I (activin A receptor type II-like kinase) 4.28 0.0083 3.09 0.0238

D50683 TGFBR2 Transforming growth factor, beta receptor II 2.09 0.0726 2.37 0.0275

NM_014733 ZFYVE16 Zinc finger, FYVE domain containing 16 1.61 0.1360 2.12 0.0138

***Toll-like receptor signaling pathway***

NM_000591 CD14 CD14 molecule 3.22 0.0496 1.57 0.5290

NM_001565 CXCL10 Chemokine (C-X-C motif) ligand 10 1.727 0.7500 17.14 0.0047

AU153366 IKBKB Inhibitor of kappa light polypeptide gene enhancer in B-cells, kinase beta 3.48 0.0049 4.22 0.0012

AF074382 IKBKG Inhibitor of kappa light polypeptide gene enhancer

in B-cells, kinase gamma  **-2.09** 0.0011 **-2.02** 0.0010

M35533 LBP Lipopolysaccharide binding protein 1.34 0.872 6.96 0.0097

AI078167 NFKBIA Nuclear factor of kappa light polypeptide gene enhancer

in B-cells inhibitor, alpha 2.32 0.0934 2.78 0.0313

AV682436 PIK3C2A Phosphoinositide-3-kinase, class 2, alpha polypeptide 2.60 0.0161 2.47 0.0150

AI679268 PIK3R1 Phosphoinositide-3-kinase, regulatory subunit 1 (p85 alpha) 3.83 0.0249 2.36 0.1320

BF740111 PIK3R4 Phosphoinositide-3-kinase, regulatory subunit 4, p150 2.71 0.0246 1.69 0.2200

BC004247 RAC1 Ras-related C3 botulinum toxin substrate 1

(rho family, small GTP binding protein Rac1) 3.11 0.0234 4.56 0.0029

BC002704 STAT1 Signal transducer and activator of transcription 1, 91kDa 6.82 0.2001 49.52 0.0077

***Wnt signaling pathway***

AI375486 APC Adenomatosis polyposis coli 13.36 0.0003 8.45 0.0008

AF275803 CACYBP Calcyclin binding protein 3.73 0.0187 6.06 0.0018

BF797381 CAMK2D Calcium/calmodulin-dependent protein kinase (CaM kinase) II delta 2.39 0.0875 3.68 0.0096

NM_004380 CREBBP CREB binding protein (Rubinstein-Taybi syndrome) 3.53 0.0017 4.69 0.0003

BF341845 CSNK1A1 Casein kinase 1, alpha 1 6.23 0.0138 7.21 0.0060

AI161318 CSNK2A1 Casein kinase 2, alpha 1 polypeptide 3.89 0.0133 4.37 0.0055

AI022089 CSNK2A2 Casein kinase 2, alpha prime polypeptide 2.04 0.0027 2.01 0.0019

NM_001320 CSNK2B Casein kinase 2, beta polypeptide 2.75 0.0482 2.63 0.0459

AA053830 CTBP1 C-terminal binding protein 1 8.33 0.0054 7.06 0.0059

NM_001904 CTNNB1 Catenin (cadherin-associated protein), beta 1, 88kDa 7.21 0.0002 7.11 0.0002

NM_003592 CUL1 Cullin 1 4.59 0.0120 4.08 0.0133

AV727101 EP300 E1A binding protein p300 2.54 0.0147 2.47 0.0120

AV700132 LONP2 Lon peptidase 2, peroxisomal 2.20 0.0040 1.80 0.0158

W61007 NFAT5 Nuclear factor of activated T-cells 5, tonicity-responsive 4.40 0.0105 4.34 0.0015

L07592 PPARD Peroxisome proliferator-activated receptor delta 3.27 0.0039 2.46 0.0137

BC000400 PPP2CA Protein phosphatase 2 (formerly 2A), catalytic subunit, alpha isoform 8.93 0.0020 9.31 0.0011

NM_004156 PPP2CB Protein phosphatase 2 (formerly 2A), catalytic subunit, beta isoform 3.58 0.0865 4.31 0.0375

NM_002717 PPP2R2A Protein phosphatase 2 (formerly 2A), regulatory subunit B (PR 52), alpha 4.78 0.0065

AA130247 PRKACB Protein kinase, cAMP-dependent, catalytic, beta 2.53 0.0411 3.01 0.0124

BC004247 RAC1 Ras-related C3 botulinum toxin substrate 1

(rho family, small GTP binding protein Rac1) 3.11 0.0234 4.56 0.0029

NM_014248 RBX1 Ring-box 1 2.51 0.0525 3.65 0.0065

BC001360 RHOA Ras homolog gene family, member A 5.20 0.0142 4.78 0.0133

NM_006930 SKP1A S-phase kinase-associated protein 1A (p19A) 12.46 0.0030 15.03 0.0012

U65019 SMAD2 SMAD family member 2 2.20 0.1550 2.88 0.0387

NM_001222 SRP72 Signal recognition particle 72kDa 5.31 0.0014 5.38 0.0008

AF267864 TBL1XR1 Transducin (beta)-like 1X-linked receptor 1 4.22 0.0025 3.58 0.0037

^a^ Mean fold changes were calculated using a division of raw expression values between experimental sample and uninfected control.

^b^ All samples were analyzed separately. Changes in gene expression with a cutoff of 2.0-fold increased or decreased expression was used and, the *p*-value was calculated

by Student's t-test. Data are displayed only where the, *p* ≤ 0.05. This *p*-value was used as a measure of the magnitude of the difference between groups and to determine

significance of the modulation. The modulated genes in the pathway are listed in alphabetic order.
